# Continuous Lower Limb Biomechanics Prediction via Prior-Informed Lightweight Marker-GMformer

**DOI:** 10.34133/cbsystems.0476

**Published:** 2026-01-15

**Authors:** Hao Zhou, Yinghu Peng, Xiaohui Li, Xueyan Lyu, Hongfei Zou, Xu Yong, Dahua Shou, Guanglin Li, Lin Wang

**Affiliations:** ^1^ Shenzhen Institutes of Advanced Technology, Chinese Academy of Sciences, Shenzhen 518055, China.; ^2^ Southern University of Science and Technology, Shenzhen 518055, China.; ^3^ School of Fashion and Textiles, The Hong Kong Polytechnic University, Hong Kong 999077, China.; ^4^ Shenzhen Lower Limb Intelligent Rehabilitation Engineering Research Center, Shenzhen 518055, China.

## Abstract

Lower limb musculoskeletal dynamics simulation has been widely used to estimate the lower limb mechanics, but challenges such as heavy reliance on force plates, poor model generalization, and high computational load hindered its application in real-time robot control systems requiring rapid feedback and inference. This study proposed the Marker-GMformer model, a marker trajectories-driven deep learning model designed for efficient and accurate continuous prediction of lower limb kinematics and dynamics. By integrating prior knowledge with global–local and spatial–temporal features from the inputted marker coordinate time series, Marker-GMformer maintained high performance while reducing computational complexity. The model also demonstrated strong generalization, accurately predicting multi-joint kinematics, moments, and ground reaction forces (GRFs) across 13 different motion patterns. The predicted results were compared to those from musculoskeletal multibody dynamics simulations and force plates. Excellent performance was achieved with average Pearson correlation coefficients (ρ≥0.97) and low root mean square errors (RMSE = 1.95° for angles, RMSE = 0.036 body weight for GRFs, and RMSE = 0.099 N·m/kg for moments) across all patterns. The findings underscored the substantial promise of the proposed method for enabling real-time monitoring of human lower limb mechanics and delivering timely feedback to optimize the control of assistive robots.

## Introduction

The dynamic analysis of the lower limb biomechanics could provide critical insights into the mechanics of gait, posture, and load distribution, which were fundamental for controlling the assistive robots, such as exoskeletons and intelligent prosthetics [1–4]. Although in vivo invasive measurements of musculoskeletal dynamics could provide direct and detailed observations of biomechanical behavior under physiological conditions, their intrusive nature, high cost, and technical complexity substantially limited their accessibility and broader application in engineering practice [5,6]. Consequently, the development of noninvasive in vivo measurement methods is of vital significance. Musculoskeletal multibody dynamics simulation (MMDS) has been developed as an efficient method to estimate the internal forces and moments [7,8]. It could integrate data from noninvasive sensors, such as motion capture (Mocap) and force plates, to simulate the mechanical behavior of bones, muscles, and tendons under different conditions, offering a noninvasive alternative to direct measurement methods [9,10].

However, some factors impeded the feasibility of MMDS for real-time control applications, where rapid, adaptive inference and immediate system responsiveness were essential. One of the factors was the dependence on force plates, as the measured ground reaction forces (GRFs) served as one of the fundamental inputs for the conventional lower limb MMDS framework. Measuring GRFs based on force plates was accompanied by several limitations that hindered practicality and accuracy. Firstly, during experiments, subjects were required to step on 2 adjacent force plates with each foot. However, due to variations in subjects’ stride lengths and the fixed length and immobility of the plates, subjects often needed to adjust their gait to ensure effective contact with the force plates. This adjustment compromised the naturalness of their gait [11]. For subjects with lower limb deformities, achieving effective contact was particularly challenging. Secondly, the requirement for force plates to be installed in laboratory environments substantially restricted their deployment in diverse settings, thereby limiting their broader applicability [12]. These limitations collectively highlighted the need for alternative or complementary methods to overcome the constraints of force plate measurements. Beyond hardware constraints, the MMDS approach was further limited by its inherently complex simulation pipelines and reliance on specialized software platforms such as OpenSim [13] and AnyBody [14]. As a result, the practical deployment of MMDS in scenarios demanding instantaneous feedback and control remained substantially challenged.

To overcome these challenges, recent studies have explored machine learning-based approaches to predict lower limb biomechanics directly from various sensing modalities, eliminating the need for force plates and complex simulation software platforms. In some of these studies, a limited number of biomechanical variables—such as single or multiple joint angles [15,16], joint moments [17], or 1- or 3-dimensional (3D) GRFs [18]—have been successfully predicted at the current or previous time steps. While these approaches showed promising potential, most of them focused on isolated components of the biomechanical system—either joint kinematics, dynamics, or GRFs—rather than providing a holistic prediction of the entire lower limb mechanical profile, which was essential for delivering comprehensive feedback in the control of assistive robots [19–21]. Moreover, although current models have achieved relatively high accuracy under restricted motion patterns—particularly for walking and running (e.g., RMSEs for joint angle as 3 to 8 [15,22,23])—most of them were limited to a narrow range of movement patterns [15–18,24]. This limitation not only prevented the validation of the proposed models’ generalization across diverse motion patterns but also fell short of meeting the requirements of assistive robotic systems that must operate across a broad spectrum of real-life functional tasks.

In terms of the proposed models, various deep learning models—such as convolutional neural networks (CNNs) [17,18,24], recurrent neural networks [16], Transformers [25], and hybrid architectures [26]—have been employed to encode input time series data (e.g., surface electromyography [sEMG] [15,16,24], inertial measurement units [IMUs] [23], GRFs [24], and marker trajectories [11,24]) and predict the biomechanics variables. However, many of these models were not structurally designed for the specific characteristics of the inputted time series data, thereby limiting their ability to effectively capture salient temporal and spatial features. In addition, some approaches lacked interpretable or physiologically grounded design rationales [18,22,26], which might pose safety concerns when deployed in safety-critical applications such as assistive robotic control. More importantly, most existing models have only been validated on a small number of motion patterns for limited target variables [11,16–18,22–26], leaving their generalization capabilities largely untested across more diverse functional movements and biomechanical outputs.

To address these limitations, this study proposed a lightweight hybrid deep learning architecture (Marker-GMformer) that leveraged marker trajectory data to continuously and efficiently predict lower limb multi-joint kinematics, joint moments, and 3D GRFs throughout the entire gait cycle across 13 distinct motion patterns. The model was specifically designed to incorporate prior knowledge of lower limb anatomical structures and to effectively capture local–global and spatial–temporal feature representations, achieving a favorable trade-off between prediction accuracy and computational efficiency. The predicted results were rigorously validated against ground-truth data obtained from traditional musculoskeletal MMDS and force plate measurements.

## Materials and Methods

### Equipment and procedure

#### Dataset

A publicly available lower limb biomechanics dataset [27], containing kinematic and GRF data from 12 healthy subjects, was used in this study. The dataset comprised various cyclic and noncyclic motion patterns, from which 13 motion patterns were selected, including walking (0.6, 1.2, and 1.8 m/s), inclined walking (5° and 10°), running (2.0 and 2.5 m/s), squatting, stair ascent/descent, step-ups (left leg), vertical jumping, and hopping. According to the specific motion instructions in Ref. [27], cyclic tasks (incline walking, walking, running, and stairs ascent/descent) generally had more repetitions due to their continuous nature, with each performed for 5 circuits (stairs ascent/descent) or 20 s, while noncyclic tasks (hopping, vertical jumping, squatting, and step-ups) had fewer repetitions, with hop performed as 10 continuous hops and the others performed with 5 repetitions. The number of samples for noncyclic tasks was relatively lower than for cyclic tasks, leading to some sample imbalance. To mitigate the potential effects of sample imbalance, 5-fold cross-validation and data augmentation techniques were employed to help reduce the risk of overfitting, which might otherwise arise from the relatively lower number of samples for some noncyclic tasks.

**Fig. 1.**
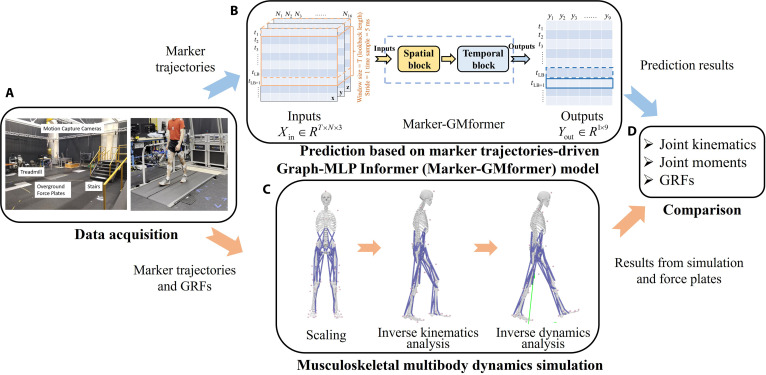
Workflows of predicting lower limb multi-joint kinematics, moments, and ground reaction forces (GRFs). (A) Experimental setup, including motion capture cameras, treadmill, force plates, and stairs, used for data acquisition [27]. (B) Deep learning-based method: marker trajectory-driven Graph-MLP Informer model. (C) Traditional method: musculoskeletal multibody dynamics simulation (MMDS). (D) Comparison of the prediction results from the Marker-GMformer model and the ground truth from MMDS.

As illustrated in Fig. 1A, marker trajectories were recorded at 200 Hz using a motion capture system equipped with 33 cameras (Vicon Ltd., Oxford, UK), while 3D GRFs were collected at 1,000 Hz using both ground force plates and an instrumented treadmill (Bertec Corporation, Columbus, OH). Detailed descriptions of how the movement patterns were performed could be found in the dataset’s supplementary material [27]. It is important to note that due to a malfunction in one of the treadmill’s force plates during data acquisition [27], GRF measurements for the right foot were unavailable in several motion patterns, including walking, incline, and running. Therefore, this study focused solely on the prediction and analysis of the left lower limb’s kinematics and dynamics.

#### Data preprocessing

The measured GRF data were downsampled to 200 Hz and temporally aligned with the marker trajectory data. Using the preprocessed marker trajectories and GRF signals as inputs, subject-specific musculoskeletal models based on the OpenSim gait2354 model [28] were employed to perform inverse kinematics and inverse dynamics simulations, yielding left lower limb joint angles and joint moments, as illustrated in Fig. 1C.

The gait2354 model is a 3D musculoskeletal model with 23 degrees of freedom, incorporating detailed lower extremity joint definitions and anthropometric parameters [28]. The model’s segment lengths, masses, and inertial properties were personalized based on each subject’s anthropometric data. Additionally, the patella was removed to avoid kinematic constraints, with quadriceps insertions represented as moving points in the tibia frame. During the inverse kinematics process, joint angles were computed by minimizing the difference between measured marker positions and model-predicted marker positions. The inverse dynamics process then calculated joint moments by applying Newton–Euler methods, using the derived joint angles and the measured GRFs. The GRFs and joint moments were normalized by body weight (BW) and body mass (N·m/kg), respectively.

**Fig. 2.**
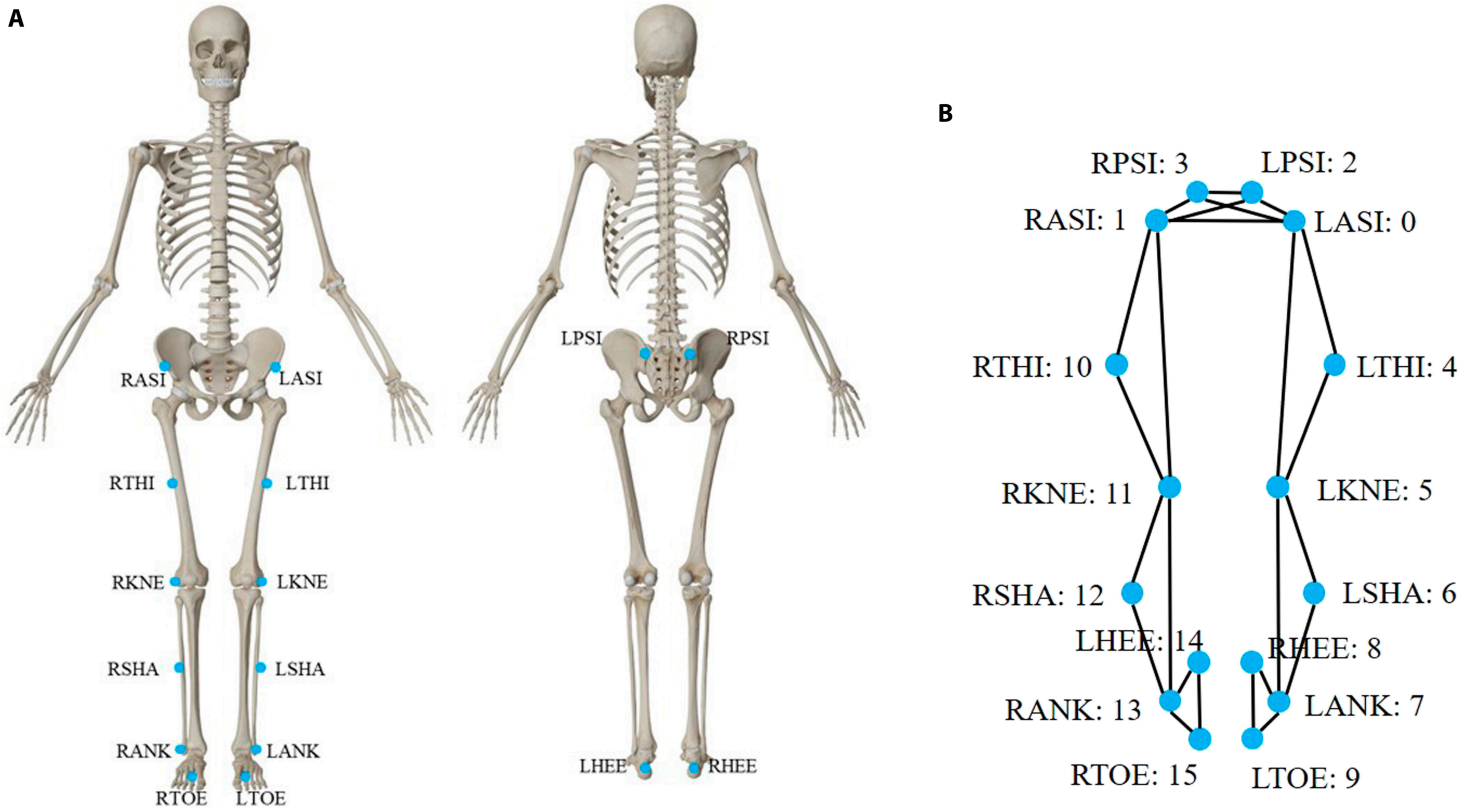
(A) A total of 16 reflective markers placed on the landmark locations of the subjects were selected. (B) The lower limb skeleton graph in physical connections used in the GCN block in Marker-GMformer, with each joint corresponding to a marker.

To prepare the data for model training and evaluation, further preprocessing was conducted. A total of 16 reflective markers were selected, shown in Fig. 2A, including RPSI, LPSI, RASI, LASI, RTHI, LTHI, RKNE, LKNE, RSHA, LSHA, RANK, LANK, LHEE, RHEE, RTOE, and LTOE. These specific marker locations were chosen to ensure comprehensive coverage of the key anatomical landmarks required for accurate lower limb motion tracking. The selection included both pelvic and lower limb joints (e.g., hips, knees, and ankles) as well as key points on the foot (e.g., heels and toes), which were essential for capturing the kinematic and dynamic behavior during various gait phases. Placing markers on these critical points could ensure reliable tracking of joint angles and lower limb motion. This marker configuration has been widely validated in similar motion capture studies and provided robust data for further biomechanical analysis [8,9,28].

The 3D spatial coordinates of these markers, along with the measured GRFs and the calculated joint angles and moments, were synchronized across time steps. After excluding invalid trials, data from all subjects and motion patterns were randomly shuffled and partitioned into training, validation, and test sets. To standardize the input features and output targets, a scaling operation was applied to each variable. The scaler was fitted exclusively on the training set, and the same scaling parameters were then used to transform all the datasets to ensure consistency across datasets.

### Prior-informed lightweight Marker-GMformer model

The workflow of the prediction method based on Marker-GMformer proposed in this study is shown in Fig. 1B. The processed 3D spatial coordinate data of 16 markers served as the inputs of Marker-GMformer, to predict the 3D GRFs, hip, knee, and ankle joint angles and moments in the sagittal plane (a total of 9 target variables) at the current time step. A 3D sliding window was used for the inputted data $X_{\text{in}}\in {\mathbb{R}}^{T\times N\times 3}$ (batch size = 1), with *T* as the window size in temporal dimension (lookback length), *N* as the number of markers (*N* = 16 in this study), and step size of the sliding window as 1 time step (5 ms). Moreover, the ground truth for model training was established using 3D GRFs from the force plates, combined with joint angles and moments calculated in OpenSim.

**Fig. 3.**
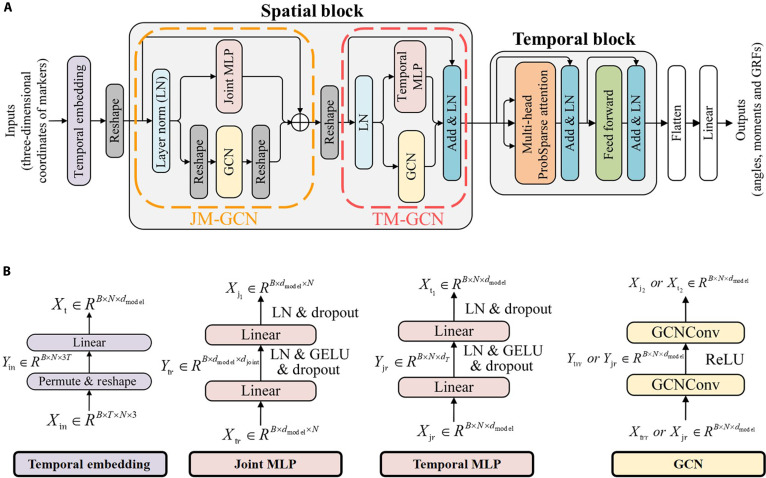
(A) The architecture of Marker-GMformer, composed of Spatial Block and Temporal Block. (B) Detailed illustration of the layers and modules in Marker-GMformer.

To efficiently extract the spatial information among anatomical landmarks embedded in the physical connections of the markers, as well as the contextual information across the temporal scale during the movements, the proposed Marker-GMformer contained a Spatial Block and a Temporal Block, as shown in Fig. 3A. Additionally, the model’s architecture also included a temporal embedding layer, a flatten layer, and a projection layer.

#### Temporal embedding layer

The temporal embedding layer was designed to embed the inputs $X_{\text{in}}\in {\mathbb{R}}^{B\times T\times N\times 3}$ (*B* was the batch size). After permuting and reshaping $X_{\text{in}}$, $Y_{\text{in}}=\text{Reshape}\left(\text{Permute}\left(X_{\text{in}}\right)\right)\in {\mathbb{R}}^{B\times N\times 3T}$. Different from the traditional embedding layer in most Transformer-based models [29–31] with the data at the same time step as a token, the temporal embedding layer transposed the inputted matrix, took the whole time series of each marker as a token, and embedded $Y_{\text{in}}$ through a fully connected layer: $X_{t}=\text{Linear}\left(Y_{\text{in}}\right)\in {\mathbb{R}}^{B\times N\times d_{\text{model}}}$.

This transposition-based feature extraction strategy has been shown to enhance the performance of Transformers in time series prediction and forecasting tasks, with excellent generalization ability across various inputted variables [32]. Moreover, it could effectively reduce computational complexity, particularly when processing long time series inputs [32,33]. This benefit arose from the fact that, regardless of the input series length, 3*T* in $Y_{\text{in}}\in {\mathbb{R}}^{B\times N\times 3T}$ would be embedded into $d_{\text{model}}$ along the temporal axis. As a result, Marker-GMformer could flexibly adapt to an input series of varying lengths (lookback length *T*).

Moreover, there was no need for position embedding, which has been commonly used in Transformers [29–31], as the position information in time series could be implicitly learned by the temporal multilayer perceptron (MLP), graph convolutional network (GCN), and feedforward neural network (FFN) modules in Marker-GMformer.

#### Spatial Block

The Spatial Block consisted of JM-GCN and TM-GCN blocks, integrating both GCN and MLP modules, to enable a fusion of local–global features informed by physical priors of human configurations [33].

$$X_{J}=\mathrm{JM}-\mathrm{GCN}\left(X_{\mathrm{tr}}\right)+X_{\mathrm{tr}}=\mathrm{JM}\left(\mathrm{LN}\left(X_{\mathrm{tr}}\right)\right)+\mathrm{GCN}{\left(\mathrm{LN}{\left(X_{\mathrm{tr}}\right)}^{T}\right)}^{T}+X_{\mathrm{tr}}$$ (1)

$$\begin{array}{c} X_{T}=\mathrm{LN}\left(\mathrm{TM}-\mathrm{GCN}\left(X_{\mathrm{Jr}}\right)+X_{\mathrm{Jr}}\right)\\ \;=\mathrm{LN}\left(\mathrm{TM}\left(\mathrm{LN}\left(X_{\mathrm{Jr}}\right)\right)+\mathrm{GCN}\left(\mathrm{LN}\left(X_{\mathrm{Jr}}\right)\right)+X_{\mathrm{Jr}}\right) \end{array}$$ (2)

where JM and TM meant the Joint MLP layer and Temporal MLP layer, respectively, LN meant layer normalization, $X_{\mathrm{tr}}$ was the reshaped output of the temporal embedding layer, $X_{J}$ was the output of JM-GCN, $X_{\mathrm{Jr}}$ was the reshaped $X_{J}$, and $X_{T}$ was the output of TM-GCN.

The GCN in both JM-GCN and TM-GCN layers leveraged prior knowledge of the physical connections among anatomical landmarks to extract local correlational information embedded in the marker coordinate time series [33]. Specifically, an adjacency matrix $A\in {\mathbb{R}}^{N\times N}$ (where *N* was the number of reflective markers) was constructed to encode the structural priors of the lower limb skeleton. As illustrated in Fig. 1, each joint node corresponded to a marker, and edges were defined according to known biomechanical linkages: for example, pelvis–hip, hip–knee, knee–ankle, and ankle–toe were connected to reflect segmental continuity. In addition, symmetric edges were added between anatomically mirrored joints (e.g., left–right hip, knee, ankle, and toe) by setting the corresponding entries in the adjacency matrix to 1. This design enabled the GCN to capture bilateral coupling and symmetry patterns in gait, beyond the local anatomical continuity. Thus, $A_{\mathrm{ij}}=1$ if marker *i* and marker *j* were anatomically or symmetrically linked, and $A_{\mathrm{ij}}=0$ otherwise.

During graph convolution, the node features (3D coordinates of each marker at a given time step) were updated as

$$H^{\left(l+1\right)}=\sigma \left({\tilde{D}}^{-1/2}{\tilde{A}\tilde{D}}^{-1/2}H^{\left(l\right)}W^{\left(l\right)}\right)$$ (3)

where $\tilde{A}=A+I$, ***I*** was an identity matrix (referring to the self-connections), $\tilde{D}$ was a diagonal matrix, ${\tilde{D}}_{\mathrm{ii}}=\sum_{j}{\tilde{A}}^{\mathrm{ij}}$, $W^{\left(l\right)}$ was the learnable weight matrix, and σ denoted the activation function (ReLU). This operation allowed each marker to aggregate information from its physically connected neighbors, thereby capturing local spatial correlations such as joint coupling and segmental coordination. In this way, the adjacency matrix served as a structural prior that guided the GCN to extract meaningful spatial interactions from the lower limb skeleton graph, rather than relying solely on data-driven correlations.

The Joint MLP layer in JM-GCN could capture and integrate interaction information across markers at each time step, while the Temporal MLP in TM-GCN could extract global contextual features from each marker’s temporal series, with the structural details shown in Fig. 2.

#### Temporal Block

It has been proven that Transformers perform exceptionally well in time series prediction [29,30,32]. In Marker-GMformer, an improved Transformer-based block was applied to extract the temporal features stored in the time series, as shown in Fig. 3. The attention mechanism could effectively capture long-range dependencies across the inputted variables, enabling information interaction among different time series. Meanwhile, based on the transposition strategy in the temporal embedding layer, the FFN and layer normalization (LN) were employed to extract and refine the internal representations within each variable’s time series, thereby enhancing the model’s ability to capture local temporal patterns and stabilize the learning process [32].

The Temporal Block adopted the ProbSparse self-attention [30] to reduce the computational complexity of the model, as the matrix multiplication operation in vanilla Transformer [31] could cause high computational complexity, especially when processing the long time series data. A sparse matrix of queries $\bar{Q}$ was used to calculate the attention values with all keys *K* in ProbSparse self-attention, where only the top-*u* queries with the largest sparsity measurement were retained for full attention computation, while the remaining queries outside the top-*u* set were approximated by attending to a global context vector. This mechanism reduced redundant query–key interactions and achieves $O\left(L\;\text{log}\;L\right)$ time complexity and memory usage, as shown in Eqs. (4) and (5) [30].

$$\text{Attention}\left(Q...K...V\right)=\text{Softmax}\left(\bar{Q}K^{T}/\sqrt{d_{\text{model}}}\right)V$$ (4)

$$\bar{M}\left(q_{i}...K\right)=\underset{j}{\text{max}}\left\{q_{i}k_{j}^{T}/\sqrt{d_{\text{model}}}\right\}-\left(1/L_{k}\right)\sum_{j=1}^{L_{k}}\left(q_{i}k_{j}^{T}/\sqrt{d_{\text{model}}}\right)$$ (5)

where $q_{i}$ and $k_{j}$ were the *i*th or *j*th row of $Q\in {\mathbb{R}}^{L_{q}\times d}$ and $K\in {\mathbb{R}}^{L_{k}\times d}$, respectively; $\bar{Q}\in {\mathbb{R}}^{u\times d}$ was a sparse matrix of ***Q***, consisting of the top-*u* queries, where $u=c\cdot \ln L_{Q}$, with constant *c* as a sampling factor (set to 3); the top-*u* queries were the $q_{i}$ corresponding to the top *u* values in the sparsity measurement $\bar{M}$; $d_{\text{model}}$ was the hidden dimension.

The FFN consists of 2 fully connected layers, with a hidden dimension of $d_{\mathrm{ffn}}=2,048$ and both input and output dimensions set to $d_{\text{model}}$. Notably, the MLP in the FFN could not only extract the variable-global features but also decode the representations to enable target variable generation [32,34–36]. The generative decoding strategy could also resolve the issue of error accumulation caused by step-by-step inference in Transformer [30].

### Configuration and performance metrics

#### Configurations

The proposed Marker-GMformer model and the other prediction models used for results comparison were trained for all the 13 motion patterns. Fivefold cross-validation was employed during training. The test set comprised 20% of the total dataset, while 20% of the remaining data was used as the validation set, and the rest was used for training. Additionally, to improve the model’s robustness and prepare it for deployment in complex scenarios, a data augmentation strategy was adopted during the model training process. Specifically, random Gaussian noise was added to the marker trajectory inputs to simulate potential signal disturbances and sensor noise. This helped the model learn to ignore irrelevant variations and focus on meaningful motion patterns.

The batch size was set to 128, and the loss function used was MSE. There were 1 Spatial Block, 1 Temporal Block, and 16 attention heads for the self-attention mechanism. The number of Markers *N* was set to 16, and the size of the sliding window *T* (lookback length) was selected in {24, 48, 72, 96} time steps, namely, {120, 240, 360, 480} ms. $d_{\text{model}}$ was 512, $d_{\text{joint}}$ was 256, and $d_{T}$ was 1,024. The activation function selected was GELU [37] and ReLU [38], and the Adam optimizer was used. The initial learning rate was 0.0001, with Cosine Annealing Scheduler applied. The early stopping mechanism was employed, with a patience of 7 epochs. The computational setup used for training and inference included an AMD Ryzen Threadripper PRO 5975WX 32-Cores 3.60 GHz Processor and an NVIDIA GeForce RTX 4090.

#### Performance metrics

The prediction results from Marker-GMformer were compared to the results obtained from the MMDS and GRFs measured by force plates. Root mean square error (RMSE), mean absolute error (MAE), and Pearson correlation coefficient (ρ) were selected as the evaluation metrics for model prediction performance. Floating point operations (FLOPs) per forward inference (batch size = 1) and the number of parameters (Params) were used to measure the models’ computational complexity. The relevant formulas were as follows:

$$\text{RMSE}=\sqrt{\frac{1}{n}\sum_{i=1}^{n}{\left(y_{i}-{\hat{y}}_{i}\right)}^{2}}$$ (6)

where $y_{i}$ was the ground truth obtained by the MMDS and ${\hat{y}}_{i}$ was the predicted value of Marker-GMformer. *n* referred to the total number of predicted time steps in the test set.

$$\mathrm{MAE}=\frac{1}{n}\sum_{i=1}^{n}\left|y_{i}-{\hat{y}}_{i}\right|$$ (7)

MAE provided a more balanced error assessment, as it did not amplify the impact of larger errors. This characteristic made MAE very useful in applications where the magnitude of prediction errors should not be excessively exaggerated.

$$\rho =\frac{\mathrm{Cov}\left(y_{i},{\hat{y}}_{i}\right)}{\sqrt{D\left(y_{i}\right)D\left({\hat{y}}_{i}\right)}}=\frac{\sum_{i=1}^{n}\left(y_{i}-\bar{y}\right)\left({\hat{y}}_{i}-\bar{\hat{y}}\right)}{\sqrt{\sum_{i=1}^{n}{\left(y_{i}-\bar{y}\right)}^{2}}\sqrt{\sum_{i=1}^{n}{\left({\hat{y}}_{i}-\bar{\hat{y}}\right)}^{2}}}$$ (8)

The Pearson correlation coefficient quantified the strength and direction of the linear relationship between 2 variables, ranging from −1 to +1. A value of +1 indicated a perfect positive linear correlation, −1 indicated a perfect negative linear correlation, and 0 denoted no linear relationship. In this study, ρ > 0.9 = excellent, ρ > 0.8 = good.

## Results

### Overall prediction performance during all the motion patterns

**Fig. 4.**
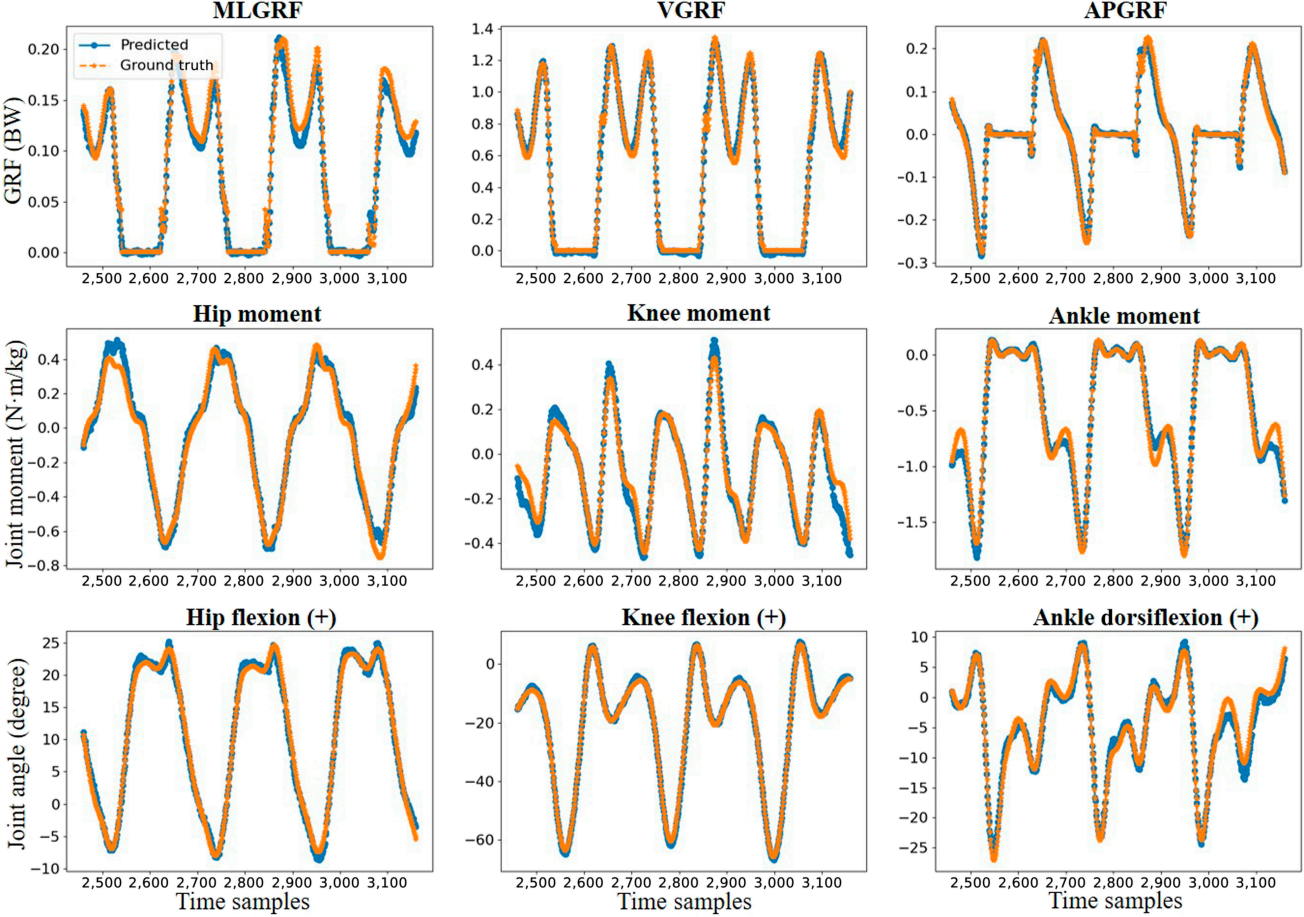
Comparison of lower limb multi-joint angles, moments, and 3-dimensional (3D) GRFs during walking at 1.2 m/s between the Marker-GMformer model (blue line) and the musculoskeletal multibody dynamics simulation (orange dots), with each subplot showing predicted vs. ground-truth curves for angles, moments, and GRFs (lookback length *T* = 48 time steps). MLGRF, APGRF, and VGRF represent mediolateral, anteroposterior, and vertical ground reaction forces, respectively.

The averaged results of the prediction of the proposed Marker-GMformer for the GRFs, multi-joint angles, and moments in the sagittal plane during all 13 motion patterns (lookback length *T* = 48) are presented in Table 1. There were excellent Pearson correlations between the predicted values and ground truth, with ρ≥0.97 for all the target variables. The average MAEs of GRFs, moments, and angles were 0.020 BW, 0.060 N·m/kg, and 1.28°, respectively. The average RMSEs for the same variables were 0.036 BW, 0.099 N·m/kg, and 1.95°, respectively. The comparison of the results from Marker-GMformer and the MMDS during walking at 1.2 m/s is shown in Fig. 4. It could be observed that the model’s predicted curves throughout the entire gait cycle closely followed the trends of the MMDS results across all target variables, demonstrating good overall agreement and indicating strong temporal modeling capability of the proposed model.

**Table 1.** The prediction results of lower limb multi-joint angles, moments, and 3-dimensional ground reaction forces (GRFs) across all the motion patterns based on different models (lookback length *T* = 48 time steps). Floating point operations (FLOPs) per forward inference and the number of parameters (Params) were used to measure the models’ computational complexity. Boldface indicates the best-performing values for each evaluation metric in the comparison.

Models	Metrics	GRFs			Joint moments			Joint angles			Computational complexity	
		MLGRF	VGRF	APGRF	Hip	Knee	Ankle	Hip	Knee	Ankle	FLOPs (M)	Params (M)
Marker GMformer	MAE	**0.0095**	**0.042**	**0.009**	**0.067**	**0.056**	**0.055**	**1.33**	**1.35**	**1.15**	73.70	3.84
	RMSE	**0.016**	**0.074**	**0.019**	**0.11**	**0.10**	**0.091**	**1.98**	**2.10**	**1.77**		
	*ρ*	**0.97**	**0.99**	**0.97**	**0.97**	**0.97**	**0.99**	**0.997**	**0.998**	**0.99**		
Informer	MAE	0.024	0.14	0.023	0.21	0.19	0.18	8.04	9.19	6.38	132.73	8.57
	RMSE	0.035	0.21	0.037	0.30	0.28	0.28	11.52	12.73	8.35		
	*ρ*	0.88	0.90	0.90	0.83	0.79	0.87	0.90	0.93	0.85		
Transformer	MAE	0.011	0.060	0.010	0.085	0.075	0.068	2.05	2.58	1.84	134.80	5.36
	RMSE	0.020	0.11	0.021	0.16	0.14	0.12	3.13	3.84	2.85		
	*ρ*	0.96	0.98	0.97	0.95	0.96	0.98	0.99	0.99	0.98		
iTransformer	MAE	0.021	0.10	0.017	0.17	0.15	0.14	5.43	5.36	3.85	208.06	4.45
	RMSE	0.030	0.16	0.028	0.24	0.21	0.21	7.48	7.46	5.22		
	*ρ*	0.91	0.95	0.94	0.91	0.89	0.94	0.96	0.97	0.94		
Crossformer	MAE	0.035	0.32	0.040	0.31	0.28	0.34	11.57	15.24	8.17	2,285.08	31.70
	RMSE	0.047	0.40	0.070	0.41	0.39	0.50	13.68	18.65	10.89		
	*ρ*	0.74	0.66	0.56	0.54	0.48	0.56	0.86	0.81	0.69		
PatchTST	MAE	0.035	0.18	0.031	0.23	0.22	0.22	12.32	14.86	7.63	610.47	2.12
	RMSE	0.049	0.24	0.045	0.30	0.30	0.29	17.19	20.10	10.21		
	*ρ*	0.71	0.88	0.82	0.78	0.74	0.86	0.76	0.76	0.73		
TimesNet	MAE	0.013	0.069	0.012	0.10	0.09	0.08	2.60	2.89	2.15	125.41	5.21
	RMSE	0.021	0.12	0.023	0.18	0.16	0.13	3.82	4.21	3.11		
	*ρ*	0.95	0.97	0.96	0.93	0.94	0.97	0.99	0.99	0.98		
DLinear	MAE	0.034	0.23	0.037	0.20	0.26	0.27	7.43	8.20	6.68	**0.005**	**0.0005**
	RMSE	0.045	0.31	0.054	0.27	0.35	0.39	9.48	10.16	8.63		
	*ρ*	0.77	0.77	0.73	0.83	0.62	0.73	0.93	0.94	0.82		

MLGRF, mediolateral ground reaction force; APGRF, anteroposterior ground reaction force; VGRF, vertical ground reaction force

Additionally, the FLOPs per forward inference and the Params of Marker-GMformer were 73.70 and 3.84 million, respectively, indicating its low computational complexity and lightweight design. The results demonstrated that the proposed prediction method could achieve high accuracy with low computational complexity, making it well-suited for real-time or resource-constrained applications, such as robotic control or real-time human gait monitoring.

### Influence of sliding-window size and stride on prediction accuracy

**Fig. 5.**
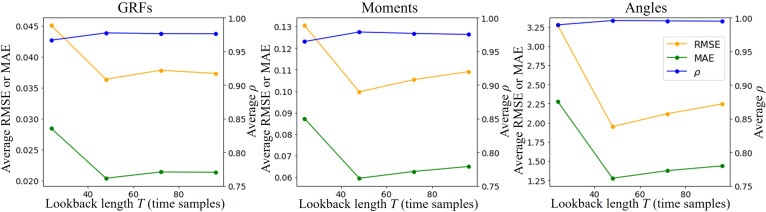
The comparison of the overall prediction performance of Marker-GMformer with different lookback lengths $T\in \left\{24,\;48,\;72,\;96\right\}$. The performance did not necessarily benefit from the increased lookback length. GRF metrics were averaged over 3D ground reaction forces; joint metrics were averaged over hip, knee, and ankle joints.

The average prediction results across all 13 motion patterns based on different sliding-window sizes (lookback length $T\in \left\{24,\;48,\;72,\;96\right\}$ time steps) are shown in Table 2. The performance metrics showed that with the increase of *T*, the prediction accuracy for all target variables initially improved, and then declined, suggesting the existence of an optimal lookback length. Shown in Fig. 5, the prediction performance reached its peak when T=48, with average RMSEs of 0.036 BW for GRFs, 0.099 N·m/kg for joint moments, and 1.95° for joint angles. The average RMSEs of GRFs, moments, and angles when T=48 were 19.3%, 28.5%, and 40.4% lower than those when T=24, while the FLOPs and Params were only 0.8% and 1.05% higher, attributed to the transposition embedding strategy in the Temporal Embedding layer. The results indicated that the prediction performance of Marker-GMformer did not exhibit a consistent positive correlation with the increase in lookback length, and the additional computational cost introduced by longer input sequences could be negligible. Therefore, considering the performance and computational complexity, a lookback length *T* = 48 was finally selected as the input sliding window size in this study.

**Table 2.** Prediction performance for lower limb joint angles, joint moments, and 3-dimensional ground reaction forces (GRFs) across all motions under different sliding-window sizes (lookback length *T*). Boldface indicates the best-performing values for each evaluation metric in the comparison.

Lookback length (steps)	Metrics	GRFs			Joint moments			Joint angles			Computational complexity	
		MLGRF	VGRF	APGRF	Hip	Knee	Ankle	Hip	Knee	Ankle	FLOPs (M)	Params (M)
24	MAE	0.013	0.060	0.013	0.10	0.079	0.086	2.32	2.46	2.04	**73.11**	**3.80**
	RMSE	0.020	0.093	0.022	0.14	0.12	0.13	3.38	3.60	2.82		
	*ρ*	0.96	0.98	0.96	0.96	0.96	0.98	0.99	0.99	0.98		
48	MAE	**0.0095**	**0.042**	**0.009**	**0.067**	**0.056**	**0.055**	**1.33**	**1.35**	**1.15**	73.70	3.84
	RMSE	**0.016**	**0.074**	**0.019**	**0.11**	**0.10**	**0.091**	**1.98**	**2.10**	**1.77**		
	*ρ*	**0.97**	**0.99**	**0.97**	**0.97**	**0.97**	**0.99**	**0.997**	**0.998**	**0.99**		
72	MAE	0.010	0.045	0.009	0.071	0.060	0.057	1.47	1.45	1.20	74.29	3.88
	RMSE	0.016	0.078	0.019	0.11	0.11	0.10	2.26	2.25	1.84		
	*ρ*	0.97	0.99	0.97	0.97	0.97	0.99	0.996	0.997	0.99		
96	MAE	0.010	0.045	0.010	0.072	0.063	0.059	1.50	1.56	1.25	74.87	3.92
	RMSE	0.016	0.076	0.020	0.12	0.11	0.10	2.33	2.44	1.96		
	*ρ*	0.97	0.99	0.97	0.97	0.97	0.99	0.996	0.997	0.99		

MLGRF, mediolateral ground reaction force; APGRF, anteroposterior ground reaction force; VGRF, vertical ground reaction force

Additionally, with lookback length fixed at *T* = 48, different sliding-window strides ({1, 12, 24, 36, 48} time steps, namely, {97.9%, 75%, 50%, 25%, 0%} overlap ratios) were evaluated. As shown in Table 3, increasing the stride yielded a gradual performance drop (higher RMSE/MAE and slightly lower ρ). The likely reason was that larger strides reduced window overlap and temporal sampling density, which increased phase mismatch between windows and lost fine-grained transition dynamics (e.g., rapid force peaks and joint moment changes), thereby weakening the model’s ability to capture fast, event-driven variations.

**Table 3.** Prediction performance for lower limb joint angles, joint moments, and 3-dimensional GRFs across all motions with different sliding-window strides (lookback length fixed at *T* = 48). Boldface indicates the best-performing values for each evaluation metric in the comparison.

Sliding window stride (time steps)	Metrics	GRFs			Joint moments			Joint angles		
		MLGRF	VGRF	APGRF	Hip	Knee	Ankle	Hip	Knee	Ankle
1	MAE	**0.0095**	**0.042**	**0.009**	**0.067**	**0.056**	**0.055**	**1.33**	**1.35**	**1.15**
	RMSE	**0.016**	**0.074**	**0.019**	**0.11**	**0.10**	**0.091**	**1.98**	**2.10**	**1.77**
	*ρ*	**0.97**	**0.99**	**0.97**	**0.97**	**0.97**	**0.99**	**0.997**	**0.998**	**0.99**
12	MAE	0.016	0.083	0.015	0.13	0.12	0.12	3.11	3.44	2.63
	RMSE	0.024	0.12	0.025	0.19	0.18	0.16	4.48	4.87	3.66
	*ρ*	0.94	0.97	0.95	0.92	0.91	0.96	0.99	0.99	0.97
24	MAE	0.018	0.090	0.016	0.14	0.13	0.13	3.38	3.82	2.81
	RMSE	0.025	0.13	0.026	0.20	0.19	0.18	4.84	5.21	3.87
	*ρ*	0.93	0.96	0.94	0.91	0.91	0.95	0.98	0.99	0.97
36	MAE	0.019	0.10	0.018	0.14	0.14	0.14	3.77	4.18	3.08
	RMSE	0.027	0.14	0.029	0.21	0.20	0.19	5.33	5.67	4.20
	*ρ*	0.92	0.96	0.93	0.90	0.90	0.94	0.98	0.98	0.96
48	MAE	0.020	0.10	0.019	0.15	0.15	0.14	3.93	4.36	3.20
	RMSE	0.028	0.14	0.030	0.21	0.21	0.19	5.49	5.87	4.43
	*ρ*	0.92	0.96	0.92	0.90	0.88	0.94	0.98	0.98	0.96

MLGRF, mediolateral ground reaction force; APGRF, anteroposterior ground reaction force; VGRF, vertical ground reaction force

### Generalization performance across the patterns

**Fig. 6.**
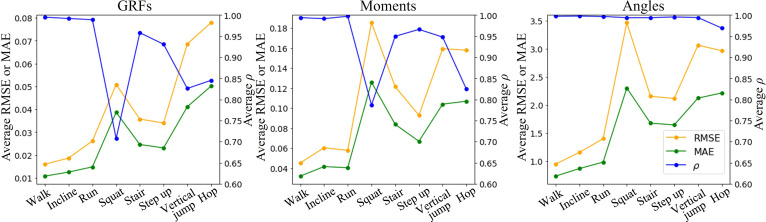
Comparison of the prediction performance of Marker-GMformer during different motion patterns, presenting strong generalization and high predictive accuracy across all motion patterns (lookback length *T* = 48 time steps). GRF metrics were averaged over 3D ground reaction forces; joint metrics were averaged over hip, knee, and ankle joints.

The prediction performance of the proposed Marker-GMformer across different motion patterns is presented in Fig. 6. Specifically, the “walking” result represented the average performance at speeds of 0.6, 1.2, and 1.8 m/s; the “incline” result corresponded to the average performance on 5° and 10° slopes; and the “running” result reflected the average performance at speeds of 2.0 and 2.5 m/s.

From the perspective of motion patterns, the model achieved the best prediction performance on walking, incline walking, and running, exhibiting excellent correlation coefficients (ρ≥0.99) and low prediction errors (RMSE of GRFs < 0.03 BW, RMSE of moments ≤ 0.06 N·m/kg, and RMSE of angles < 1.5°). In contrast, the prediction accuracies for patterns such as squatting, vertical jumping, and hopping were relatively lower, with squat being the most challenging (average ρ for GRFs and moments in squat were 0.71 and 0.79, respectively). From the target variables perspective, the reduced prediction accuracies in squat, vertical jump, and hop were primarily attributed to the APGRF and hip moment, where the average ρ of these 2 variables ranged from 0.50 to 0.78, shown in Tables S4, S7, and S8. In comparison, the prediction performance for the remaining target variables remained strong, with ρ ranging from 0.84 to 0.98. Moreover, among all predicted variables, Marker-GMformer exhibited the best generalization performance across all the motion patterns in kinematics variables (ρ>0.95), while showing relatively weaker generalization in some dynamics variables.

Additionally, to test whether gains were statistically meaningful, paired 2-sided *t* tests were performed at the motion level (*n* = 13 motions). Marker-GMformer was compared with each baseline model in Table 1 using the 13 paired motion values ($\Delta ={\text{RMSE}}_{\text{ours}}-{\text{RMSE}}_{\text{baseline}}$). Results in Figs. S5 to S7 showed consistent, significant reductions across all GRF variables (all *P* ≤ 0.0235) and joint angles (all *P* ≤ 0.0100), and mostly significant gains for joint moments.

In general, the model exhibited strong generalization and high predictive accuracy across all motion patterns, with RMSEs of GRFs < 0.08 BW, moments < 0.19 N·m/kg, and angles < 3.5°, indicating its robust adaptability and stability.

### Ablation study

This study conducted a comprehensive ablation study, which included 2 parts: A comparative analysis between the proposed model and other state-of-the-art models to validate its overall performance advantages, and a series of component removal experiments to assess the contribution of each module to the final performance, thereby providing deeper insights into the effectiveness of the model architecture.

To ensure a consistent and fair evaluation, the hyperparameter settings of the compared models were aligned as closely as possible with those of Marker-GMformer. Specifically, the models used for comparison were configured as follows. iTransformer [32] and TimesNet [39] were implemented with 2 layers, while Transformer [31], Informer [30], Crossformer [40], and PatchTST [29] were implemented with 2 encoder layers and 1 decoder layer. To ensure fairness, all Transformer-based models shared the same set of key hyperparameters, including batch size, number of attention heads, learning rate, lookback length, and hidden dimension $d_{\text{model}}$, so that differences in performance could be attributed to model design rather than inconsistent settings. Additionally, in TimesNet [39], $d_{\text{model}}$ = 64 and the periodic interval *k* = 5 during Fast Fourier Transform. Shown in Table 1, compared to other models such as Transformers (Transformer [31], Informer [30], iTransformer [32], Crossformer [40], and PatchTST [29]), CNNs (TimesNet [39]), and Linear models (DLinear [36]), Marker-GMformer achieved a superior balance between prediction performance and computational complexity. Marker-GMformer demonstrated more accurate prediction across all dynamic and kinematic target variables while maintaining substantially fewer parameters and FLOPs. Although DLinear [36] exhibited much lower computational complexity than the compared models, its prediction errors were substantially higher and failed to meet the accuracy requirements for lower limb biomechanics estimation.

The results of the component removal experiments are summarized in Table 4. It could be observed that removing any of the key modules—whether the Temporal Block, the entire Spatial Block, or its subcomponents (JM-GCN or TM-GCN)—would lead to a noticeable drop in prediction performance. This indicated that each module contributed positively to the effectiveness of Marker-GMformer. Notably, the degradation in performance caused by removing the Spatial Block, either entirely or partially, was more substantial than that caused by removing the Temporal Block. This might be attributed to the fact that the Spatial Block not only captured the local–global spatial features among markers but also encoded prior knowledge about the anatomical structure of the human lower limbs.

**Table 4.** Ablation study on the impact of component removal (lookback length *T* = 48 time steps). GRF metrics were averaged over 3-dimensional ground reaction forces; joint metrics were averaged over hip, knee, and ankle joints. Boldface indicates the best-performing values for each evaluation metric in the comparison.

Spatial block		Temporal block	Metrics	GRFs	Joint moments	Joint angles
JM-GCN	TM-GCN					
**×**	**√**	**√**	MAE	0.043	0.15	4.83
			RMSE	0.064	0.21	6.49
			*ρ*	0.94	0.91	0.96
**√**	**×**	**√**	MAE	0.022	0.065	1.52
			RMSE	0.038	0.10	2.23
			*ρ*	0.98	0.98	0.995
**√**	**√**	**×**	MAE	0.021	0.065	1.38
			RMSE	0.037	0.10	2.09
			*ρ*	0.98	0.98	0.995
**×**	**×**	**√**	MAE	0.029	0.10	2.69
			RMSE	0.047	0.15	3.88
			*ρ*	0.96	0.95	0.98
**√**	**√**	**√**	MAE	**0.020**	**0.060**	**1.28**
			RMSE	**0.036**	**0.099**	**1.95**
			*ρ*	**0.98**	**0.98**	**0.995**

Further subdivision results across joint angles, joint moments, and GRFs are summarized in Table S9. The prediction of joint angles was most affected, with the average RMSE of hip, knee, and ankle joints increasing by 7.2% to 233% after component removal. In contrast, the prediction of GRFs was relatively less sensitive, with the average RMSE of the 3D GRFs changing by only 2.8% to 78%, while joint moments showed intermediate sensitivity. These results confirmed that both the Spatial Block and the Temporal Block contributed to improving performance across all categories of output variables.

### Model interpretability

To elucidate what Marker-GMformer learned beyond aggregate accuracy, post-hoc interpretability analyses were conducted on its 2 key components: the GCN layer in Spatial Block and the ProbSparse self-attention layer in Temporal Block. The goal was to verify that the model’s internal representations aligned with physiologically meaningful dependencies—local skeletal coupling in space and selective long-range interactions in time—rather than exploiting dataset idiosyncrasies.

#### GCN layer

**Fig. 7.**
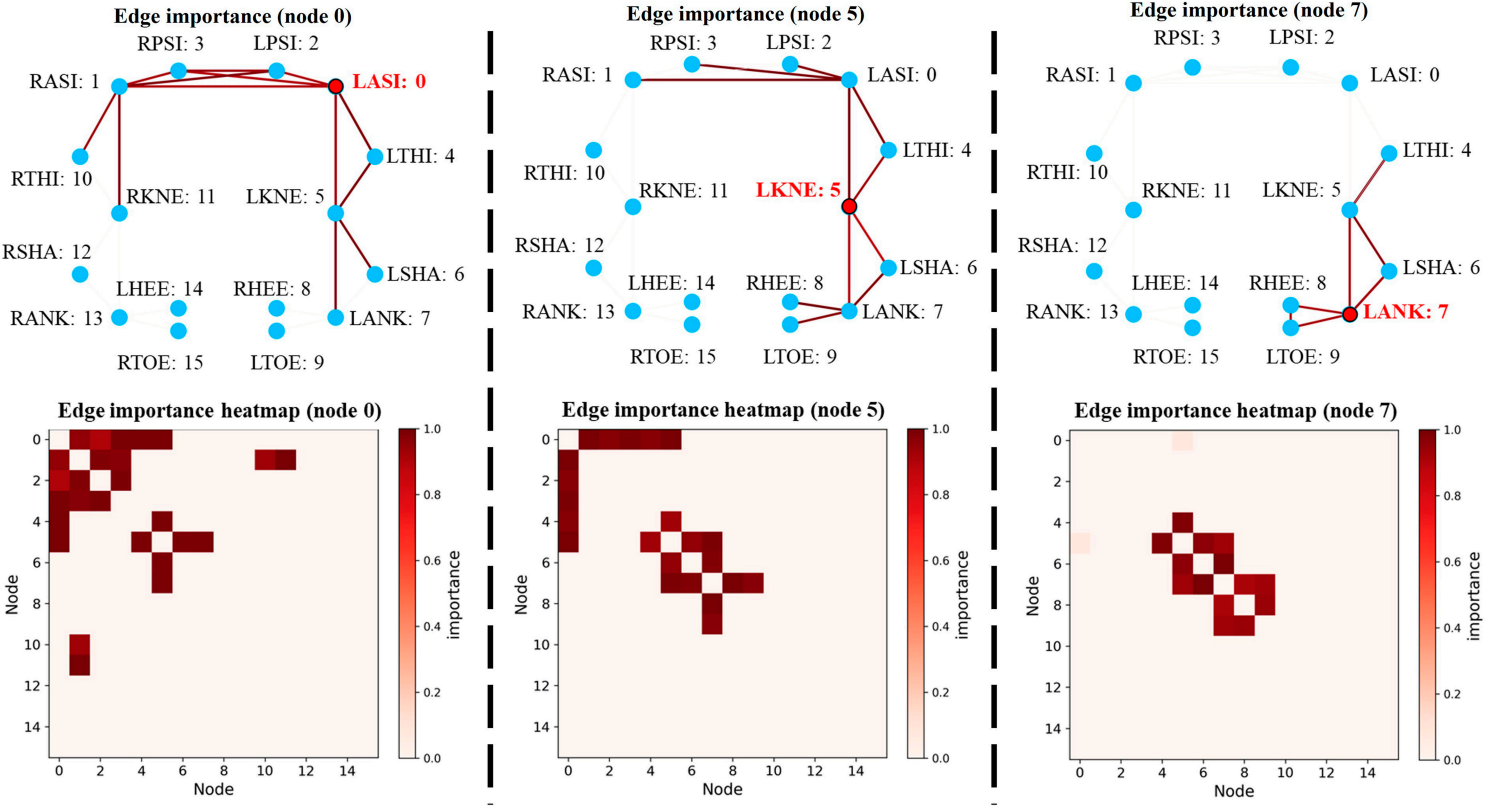
GNNExplainer-based interpretability of the JM-GCN on a representative walking trial. Top: edge-importance overlays for target nodes LASI (node 0), LKNE (node 5), and LANK (node 7); darker and thicker edges indicate higher contribution (normalized 0 to 1). Bottom: corresponding edge-importance heatmaps (rows/columns are node indices).

We analyzed the GCNs in the Spatial Block using GNNExplainer [41], which learned a soft mask over graph edges (and optionally node features) to maximize the mutual information between the GCN’s output for a target node and the masked subgraph. The resulting mask provided a normalized edge importance score in [0,1], indicating how much each connection contributed to the node’s representation. Focusing on the GCN layer in JM-GCN, we selected 3 anatomically critical left-leg markers—LASI (node 0), LKNE (node 5), and LANK (node 7)—and generated explanations on a representative walking trial from one subject. The visualizations (edge-importance overlays on the lower limb skeleton and corresponding heatmaps, shown in Fig. 7) showed a clear, physiologically consistent pattern: For LASI (pelvis), edge importance concentrates on the pelvic ring (LASI–RASI–LPSI–RPSI) and its immediate connectors, consistent with the pelvis acting as a rigid hub anchoring both limbs; for LKNE (knee), salient edges cluster along the ipsilateral chain LTHI–LKNE–LSHA–LANK, with moderate support from proximal pelvic links, capturing knee coupling with the thigh and shank; for LANK (ankle), importance peaks around distal shank–foot connections (LSHA–LANK–LHEE/LTOE), matching the expected local mechanics governing ankle motion and foot contact.

Across nodes, the heatmaps showed a clear block-diagonal pattern: strong same-side clusters and weaker cross-side links (pelvis aside). This indicated that the GCN favored local anatomical correlations while preserving bilateral topology, confirming that the Spatial Block learned physiologically meaningful spatial features.

#### ProbSparse self-attention layer

**Fig. 8.**
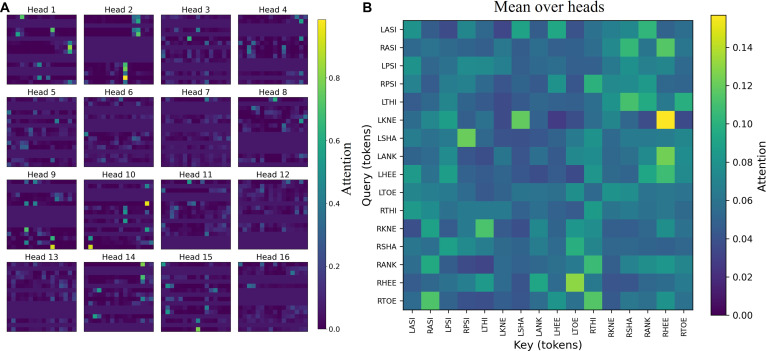
ProbSparse attention weights in the Temporal Block. (A) per-head self-attention maps for all 16 heads on a representative walking sample (rows = queries, columns = keys; higher intensity = larger attention). Many heads exhibit sparse focusing, concentrating mass on only 2 to 3 key nodes. (B) head-mean attention heatmap with joint names on both axes, highlighting a subset of nodes with stable global influence.

We further examined the Temporal Block by visualizing the ProbSparse self-attention weights for all 16 heads and the head-mean map (Fig. 8). Although this attention operated after the Spatial Block—thus receiving highly nonlinear node embeddings—it still exhibited the hallmark sparse focusing behavior: many heads concentrated most of their mass on only 2 to 3 key nodes. The specific nodes emphasized vary across heads, indicating functional diversity among heads and suggesting complementary roles (e.g., some heads consistently emphasized pelvis/thigh nodes, whereas others prioritized knee, ankle, or foot nodes). This head-wise specialization enabled the model to select a small set of representative nodes and broadcast their information over the entire lookback window, thereby capturing global temporal trends and long-range dependencies without diffusing attention.

The head-mean heatmap highlighted a subset of nodes with stable global influence across heads, consistent with the ProbSparse design objective of retaining the most informative queries–keys while discarding low-value interactions. Together with the GCN’s local spatial aggregation, these patterns showed that the Temporal Block contributed selective, time-oriented integration that complemented the local anatomy-aware features, improving efficiency and interpretability simultaneously.

### Deployment results

To further evaluate the practical feasibility of the proposed model in real-time scenarios, the trained model was deployed on both a laptop and an embedded device. On the laptop equipped with an Intel(R) Core(TM) i7-14650HX CPU (2.20 GHz, 16 cores, 24 threads) and an NVIDIA GeForce RTX 5060 Laptop GPU, the model achieved an average inference time of 2.80 ms per forward inference (batch size = 1). On the embedded platform, Jetson Xavier NX (8 GB, 69.6 mm × 45 mm form factor, 21 TOPS INT8 peak performance, 10 to 20 W typical power envelope), the average inference time was 12.51 ms per forward inference under the same settings. These results demonstrated that the model could already operate in real time on desktop-class hardware, and that it reached near-real-time performance on the Xavier NX. Given the substantially higher computational capacity of newer Jetson Orin NX (>117 TOPS) and AGX Orin (>200 TOPS) devices, it was expected that the inference latency would be further reduced to within a few milliseconds (<5 ms) per sample when deployed on these platforms.

## Discussion

In this study, a marker trajectories-driven method was proposed for the prediction of lower limb mechanics and GRFs, avoiding the dependence on force plates and MMDS. With markers’ 3D coordinates as inputs, the multi-joint angles, moments, and 3D GRFs of the left lower limb could be accurately and continuously predicted by the proposed Marker-GMformer model, with low computational complexity and efficient inference. Excellent average Pearson correlations, low average MAEs, and average RMSEs were presented in the prediction results for all of the target variables across 13 motion patterns, which was comparable to the results of the MMDS. The proposed method could be effectively applied to the real-time control of assistive robots, such as exoskeletons and intelligent prostheses, as well as the real-time monitoring and assessment of lower limb biomechanics.

The integration of prior anatomical knowledge, the innovative use of spatiotemporal feature extraction, and the lightweight architecture made Marker-GMformer a unique and efficient solution for lower limb biomechanics prediction. By explicitly encoding anatomical knowledge into the model through a structured graph, Marker-GMformer captured local and global features of the marker coordinate data in a physiologically meaningful way [33,42–44]. This model contrasted with some other models based on IMU [23] and sEMG [15,16,22,25], which typically relied on simplified assumptions and did not incorporate prior anatomical knowledge, limiting their ability to generalize across different individuals or motion patterns. Recent studies have also applied physics-informed neural networks (PINNs) to biomechanics modeling, such as SSPINNpose [45] and physics-informed CNN [22]. While these approaches improved interpretability by embedding biomechanical equations, their reliance on idealized physical formulations and validation on narrow motion ranges constrained their applicability to complex 3D or high-dynamic tasks. In contrast, Marker-GMformer learned biomechanical dependencies implicitly through data-driven anatomical priors, enabling accurate and scalable prediction across diverse motion patterns. In addition to its anatomical prior integration, Marker-GMformer incorporated the ProbSparse self-attention mechanism [30], which reduced computational complexity by sparsely attending to the most relevant parts of the input. The transposition embedding strategy [32,33] further enhanced the model’s ability to process variable-length time-series data while maintaining spatial coherence. This strategy also enabled the model to handle long-range dependencies across time more effectively [32]. Together, these innovations reduced the computational load, enabling the model to achieve high performance with lower resource requirements, which demonstrated the proposed method’s suitability for real-time applications and edge deployment.

The proposed framework provided practical advantages over traditional MMDS pipelines, particularly in applications that require real-time feedback and rapid inference. By leveraging only marker trajectories as input, it eliminated the dependency on force plates and biomechanical modeling platforms such as OpenSim [28] or AnyBody [14]. In doing so, the method helped to promote noninvasive and scalable real-time gait analysis, circumventing the need for some complex hardware or subject-specific modeling procedures [46]. Notably, shown in Table 5, compared to the data recorded by wearable sensors in some studies, such as IMU [23] and sEMG [15,16,22,25], marker trajectory data obtained through optical motion capture system generally exhibited higher spatial fidelity and lower noise [47], contributing to the improved accuracy and generalization performance across the 13 motion patterns observed in this study. Moreover, Marker-GMformer could simultaneously predict a comprehensive set of 9 biomechanical target variables, encompassing joint angles, joint moments, and GRFs across the 13 motion patterns, demonstrating strong generalization. Many previous studies were restricted to isolated tasks—predicting only kinematics or GRFs [11,15,16,22]—or were limited to specific gait phases, such as stance phase [23,48,49], or a narrow range of motion patterns, typically walking and running [23,25,50]. In contrast, the unified architecture presented here supported continuous prediction of both kinematics and dynamics variables across complete gait cycles.

**Table 5.** The comparison of the proposed method with related studies focused on the lower limb mechanics prediction.

Ref.	Sensors	Models	Predicted variables	Prediction performance
Zhong et al. [15]	sEMG	Adaptive network-based fuzzy inference system (ANFIS)	Knee joint angle during walking at 5 different speeds.	RMSE: 8.02° ± 2.29° *ρ*: 0.92 ± 0.05
Lu et al. [16]	sEMG	CNN-LSTM	Hip, knee, and ankle angles during 7 movements.	RMSE: 6.09° ± 2.12°
Skals et al. [11]	Kinematic data (Mocap)	Musculoskeletal model	GRFs during 5 different movements.	RMSE: Moments: 0.018 BW·BH GRFs: 0.07 BW *ρ*: Moments: 0.92 GRFs: 0.83
Zhang et al. [22]	sEMG	CNN	Knee joint angle during walking at 4 different speeds.	RMSE: 5.42° *ρ*: 0.99
Xiang et al. [23]	Angular velocity and acceleration (IMU)	LSTM-MLP	Ankle joint angle, moment, and JCFs during walking and running.	RMSE: Angle: 3.09° JCFs: 0.10–0.52 BW Moments: 0.155 N·m/kg
Proposed method	Marker trajectories (Mocap)	Marker-GMformer	Hip, knee, and ankle angles, moments, and GRFs during 13 motion patterns.	RMSE: Angle: 1.95° Moments: 0.099 N·m·kg^-1^ GRFs: 0.036 BW *ρ*: Angle: 0.98° Moments: 0.98 GRFs: 0.99

sEMG, surface electromyography; IMUs, inertial measurement units; Mocap, motion capture; BW·BH, subject’s body weight multiplied by their body height

Beyond the overall model performance, we observed task- and variable-specific disparities in prediction accuracy. Motions characterized by high variability or dynamic transitions—such as squatting, vertical jumping, and hopping—yielded relatively lower correlations with the ground truth, particularly for APGRF and hip moment. Through systematic analysis, we have identified several interconnected factors contributing to these prediction errors. From a data perspective, these motions were inherently associated with greater intra-subject variability, less consistent movement patterns, and more complex contact dynamics [51]. This led to noisier input signals and reduced reproducibility across samples [52], which challenged the model’s ability to generalize. Additionally, the relatively short contact time with the ground during tasks such as hopping and jumping resulted in sharper force transitions, making it difficult for the model to precisely capture the force peaks and moment changes [53]. From a model perspective, although Marker-GMformer could effectively capture spatial–temporal dependencies in structured motion, it might struggle with highly nonlinear or abrupt biomechanical events [54]. Furthermore, APGRF prediction challenges arose from these forces’ greater sensitivity to subtle changes in movement strategy and center-of-mass trajectory variations, while hip joint moments demonstrated increased prediction errors due to the complex multi-planar muscle coordination required during dynamic movements. To address these issues, several potential remedies could be explored in future work. From a modeling perspective, introducing task-specific sub-models or mixture-of-experts architectures could help the network specialize in high-dynamic tasks [55], improving adaptation to motion-dependent force and moment characteristics. Incorporating physics-informed constraints or biomechanical priors—for instance, enforcing smooth force and moment transitions, symmetry constraints, or energy consistency—may also enhance physical plausibility during dynamic movements. From a data perspective, augmenting the training set with additional samples from high-intensity or noncyclic motions could improve robustness and coverage of extreme dynamics.

Additionally, we also investigated the influence of lookback length *T* on prediction performance, shown in Fig. 5. It was found in previous studies [32,33] that the prediction performance of Spatial Block and Transformers with transposition embedding strategy (Temporal Block) could improve as the lookback length *T* increased, but the impact of varying the *T* on model prediction performance remained uncertain when the Spatial Block and Temporal Block were integrated. It was crucial to choose an appropriate lookback length by comprehensively considering the performance of the model and its computational complexity. Therefore, the impact of different lookback lengths on the prediction performance was analyzed in this study. Unlike the results in previous studies [32,33] and naive assumptions that longer time windows could yield better outcomes, the results in Fig. 5 indicated that there existed an optimal inputted time series length (*T* = 48) in this study. Excessive lookback lengths would introduce redundancy and dilute salient features, while too-short windows might hinder temporal feature extraction [30,56]. This nonmonotonic behavior contrasted with prior observations in time series modeling [32,33], and might be attributed to the nonlinear interaction between the Spatial Block and Temporal Block in Marker-GMformer. The fusion of these 2 components, each extracting hierarchical features from spatial and temporal domains, respectively, could lead to overfitting or information saturation when the input sequence became unnecessarily long [30,36,56]. This highlighted the importance of selecting an appropriate temporal window that balanced information richness with model stability and generalization.

The proposed method showed strong potential for application in indoor rehabilitation scenarios and laboratory-based validation or prototyping of wearable assistive devices, such as exoskeletons. However, the method relied on optical motion capture systems, which restricted its use to controlled laboratory environments where precise and noise-free data acquisition was feasible. This dependence limited its direct applicability in more dynamic or uncontrolled real-world settings. To enhance its translational potential for real-world use in exoskeletons or prostheses, the framework can be extended to portable markerless motion capture systems [57,58], including vision-based and IMU-based approaches. In the vision-based setting, human lower limb anatomical key points can be estimated from video data using pose estimation algorithms [33,59]; the predicted 3D coordinates of these key points can then replace the reflective marker coordinates as input to Marker-GMformer. In the IMU-based setting, wearable IMU sensors placed on the pelvis, thighs, shanks, and feet can serve as the nodes of the lower limb graph, with their 9-axis signals (accelerometer, gyroscope, and magnetometer) used as node features. The GCN can thus be reconstructed with IMU-defined nodes and anatomical linkages as edges, enabling the network to extract spatial dependencies from sensor signals. Both approaches allow the existing architecture to remain largely unchanged, requiring only an input adaptation layer, and make it possible to deploy the model in more dynamic, unconstrained environments.

There are several limitations to this study. The dataset used contained recordings from only 12 healthy participants, which may limit the generalizability of the proposed model to populations with gait impairments such as stroke survivors and elderly individuals. Moreover, due to dataset constraints, predictions were restricted to the left leg. For healthy and elderly subjects with generally symmetrical gait, extending the framework to bilateral prediction can be achieved by modifying the model output to predict bilateral kinematics and dynamics simultaneously. In contrast, for patients with unilateral impairments (e.g., hemiplegia), gait asymmetry poses additional challenges, and further domain adaptation or fine-tuning with pathological datasets will be required to ensure accurate bilateral prediction. Future work will expand the dataset to include impaired-gait subjects and further validate the model’s bilateral prediction accuracy to provide a more comprehensive assessment of inter-limb coordination.

## Conclusion

In this study, Marker-GMformer was proposed, a marker trajectories-driven framework for efficient and accurate continuous prediction of lower limb multi-joint kinematics, joint moments, and 3D GRFs. Marker-GMformer integrated prior knowledge of lower limb anatomical structure, jointly captured temporal and spatial features, and adopted a lightweight architectural design. Across 13 motion patterns, the prediction results showed strong agreement with the outputs of MMDS, confirming the reliability of the approach. Beyond accuracy, the model achieved this performance with low computational complexity and short inference latency, underscoring its suitability for real-time applications. The method could thus support real-time monitoring of lower limb mechanics, and provide a solid foundation for instantaneous kinematic and dynamic feedback in the control of assistive robotic systems such as exoskeletons and intelligent prostheses.

## Data Availability

The data used to support the findings of this study are available from the corresponding author upon reasonable request.
