# Supplementary material for: Continuous Lower Limb Biomechanics Prediction via Prior-Informed Lightweight Marker-GMformer
Source: Cyborg Bionic Syst. 2026 Jan 15;7:0476. doi: 10.34133/cbsystems.0476 (PMC12804596; doi:10.34133/cbsystems.0476)
Supplement: Supplementary 1 — Supplementary Text Figs. S1 to S7 Tables S1 to S9 [file cbsystems.0476.f1.docx]

**Supplementary Materials and Methods**

**Simulation Validation of Exoskeleton-Assisted Control**

To further verify the practical applicability of the proposed Marker-GMformer model for exoskeleton-assisted control, a preliminary simulation study was conducted in OpenSim [1]. Using the walking motion data of one representative subject as an example, the simulation aimed to evaluate the potential assistive effects of the exoskeleton by introducing knee joint assistance torques derived from the model’s predicted joint moments. Specifically, the muscle activations of a bilateral lower-limb musculoskeletal model were compared between baseline (without exoskeleton) and exoskeleton-assisted conditions to assess the reduction in muscle effort. The entire simulation framework was implemented using the OpenSim Moco toolbox [2], formulated as an optimal control problem with muscle activations and exoskeleton torques as control variables.

**A. Musculoskeletal model and experimental data**

A planar musculoskeletal model of the lower extremity Gait10dof18musc [1] was employed in this study, as shown in Figure S1. The model consisted of 10 degrees of freedom (DOF) representing pelvis translation (2 DOF), pelvis tilt (1 DOF), hip flexion (bilateral, 2 DOF), knee flexion (bilateral, 2 DOF), ankle dorsiflexion (bilateral, 2 DOF), and lumbar extension (1 DOF). The model included 18 Hill-type muscle-tendon actuators representing major lower limb muscle groups. To model foot–ground interaction, a set of compliant Hunt–Crossley contact elements were placed on each foot—centered at the heel, the first/third/fifth metatarsal heads, the hallux, and the lesser toes—so that the contact patches spanned the typical high-pressure regions encountered during stance.

Reference kinematic data were obtained from experimental gait analysis, including joint angles and angular velocities for a complete gait cycle. The musculoskeletal simulation problem was formulated as an optimal control problem using the direct collocation method implemented in OpenSim Moco [2].

**B. Baseline gait tracking without exoskeleton**

The baseline tracking problem was formulated using MocoTrack, minimizing the weighted sum of tracking error and muscle effort:

$$J_{\text{baseline}}=w_{s}\int_{t_{0}}^{t_{f}} \parallel q(t)-q_{\text{ref}}(t)\parallel^{2}\text{ }dt+w_{c}\int_{t_{0}}^{t_{f}} \parallel a(t)\parallel^{2}\text{ }dt+w_{p}\sum_{i} ((x_{i}^{\text{left}}(t_{0})-x_{i}^{\text{right}}(t_{f}))^{2}+(x_{i}^{\text{right}}(t_{0})-x_{i}^{\text{left}}(t_{f}))^{2})$$

where *J* was the objective functional, *q(t)* denoted the generalized coordinates (joint angles), $q_{\text{ref}}(t)$ were the reference coordinates from experimental data, *a(t)* denoted the muscle activations (controls), $x(t)\in\mathbb{R}^{n}$ represented the state vector (joint angles, velocities, and muscle activations), *w_s_* was the state tracking weight (set to 10.0), *w_c_* was the control effort weight (set to 5.0), and *w_p_* was the periodicity weight (set to 1.0).

**C. Exoskeleton-assisted gait with torque tracking**

Knee exoskeleton assistance was modeled using coordinate actuators applied to bilateral knee joints. The exoskeleton torques $\tau_{\text{exo}}^{r}(t)$ and $\tau_{\text{exo}}^{l}(t)$ for right and left knees were added to the system dynamics:

$$M(q)\ddot{q}+C(q,\dot{q})+G(q)=\tau_{\text{muscle}}(a,q,\dot{q})+\tau_{\text{exo}}(t)+\tau_{\text{ext}}$$

The exoskeleton-assisted gait was formulated using MocoStudy with four simultaneous objectives:

$$J_{\text{exo}}=w_{1}J_{\text{state}}+w_{2}J_{\text{control}}+w_{3}J_{\text{effort}}+w_{4}J_{\text{symmetry}}$$

1) State tracking objective: $J_{\text{state}}=\int_{t_{0}}^{t_{f}} \parallel q(t)-q_{\text{ref}}(t)\parallel^{2}\text{ }dt$

2) Control tracking objective (exoskeleton torque tracking): $J_{\text{control}}=\int_{t_{0}}^{t_{f}} \parallel\tau_{\text{exo}}(t)-\tau_{\text{exo}}^{\text{ref}}(t)\parallel^{2}\text{ }dt$, where $\tau_{\text{exo}}(t)=[\tau_{\text{exo}}^{r}(t),\tau_{\text{exo}}^{l}(t)]^{T}$ represented the exoskeleton assistance torques, and $\tau_{\text{exo}}^{\text{ref}}(t)$ was the prescribed exoskeleton torque profile.

3) Control effort objective: $J_{\text{effort}}=\int_{t_{0}}^{t_{f}} \parallel a(t)\parallel^{2}\text{ }dt$

4) Periodicity objective: $J_{\text{symmetry}}=\sum_{i} ((x_{i}^{\text{left}}(t_{0})-x_{i}^{\text{right}}(t_{f}))^{2}+(x_{i}^{\text{right}}\left( t_{0} \right)-x_{i}^{\text{left}}\left( t_{f} \right))^{2})$

The weights were set as: w_1_ = 10.0, w_2_ = 10.0, w_3_ = 5.0, w_4_ = 1.0.

The reference exoskeleton torque profile $\tau_{\text{exo}}^{\text{ref}}(t)$ was designed to provide assistance during the stance phase and facilitate swing initiation with 20% of the predicted knee moments outputted by Marker-GMformer model. The continuous optimal control problem was transcribed into a nonlinear programming (NLP) problem using Hermite-Simpson direct collocation. The NLP was solved using the interior-point optimizer IPOPT [3]. The simulation framework was implemented in MATLAB R2024a using OpenSim 4.5 and Moco 0.4.0. All computations were performed on a workstation with Intel Core i9-13900H CPU (2.6 GHz) and 64 GB RAM.

**Supplementary Results**

**Results of Exoskeleton-Assisted Simulation**

The simulation results revealed that knee exoskeleton assistance markedly affected lower-limb muscle activation while maintaining stable joint kinematics and accurate torque tracking.

As shown in Figure S2, the application of exoskeleton assistance resulted in a pronounced reduction in overall muscle activation of the right leg. The mean activation across nine major lower-limb muscles decreased from 0.0745 in the baseline (without exoskeleton) condition to 0.0513 with assistance, corresponding to a 31.12% decrease in total muscular effort. This decline was especially evident in the hamstrings, biceps femoris short head (bifemsh), rectus femoris, vasti, gastrocnemius, and iliopsoas, which are primarily responsible for hip flexion, knee flexion, and knee extension. These muscles play key roles during the stance and propulsion phases of gait, indicating that the applied assistive torque effectively offloaded knee joint demands and reduced the need for active muscle force generation.

The effectiveness of the applied torque tracking was shown in Figure S3, where the actual exoskeleton torques closely followed the reference torque profiles predicted by Marker-GMformer, achieving low RMSE values of 0.0073 Nm (right knee) and 0.0087 Nm (left knee). Furthermore, as illustrated in Figure S4, the hip, knee, and ankle joint angles under exoskeleton assistance remained consistent with those in the reference condition (RMSE ≤ 6.5°), confirming that the assistance preserved the natural gait motion while effectively reducing muscular effort.

These results demonstrated that the Marker-GMformer–based exoskeleton torque provided accurate and physiologically aligned assistance, efficiently reducing the activation of key muscle groups while maintaining natural lower-limb kinematics.


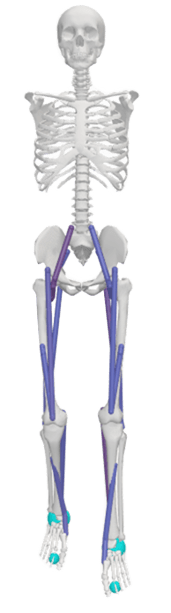

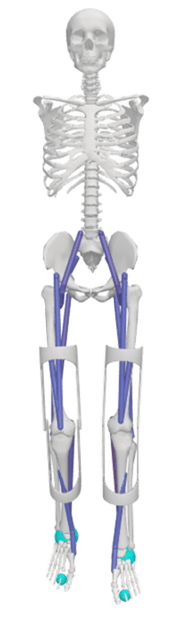


(A) (B)

Figure S1: Planar musculoskeletal model used in the simulation. (A) Baseline model without exoskeleton. (B) Model with bilateral knee exoskeleton actuators providing assistive torques during gait.


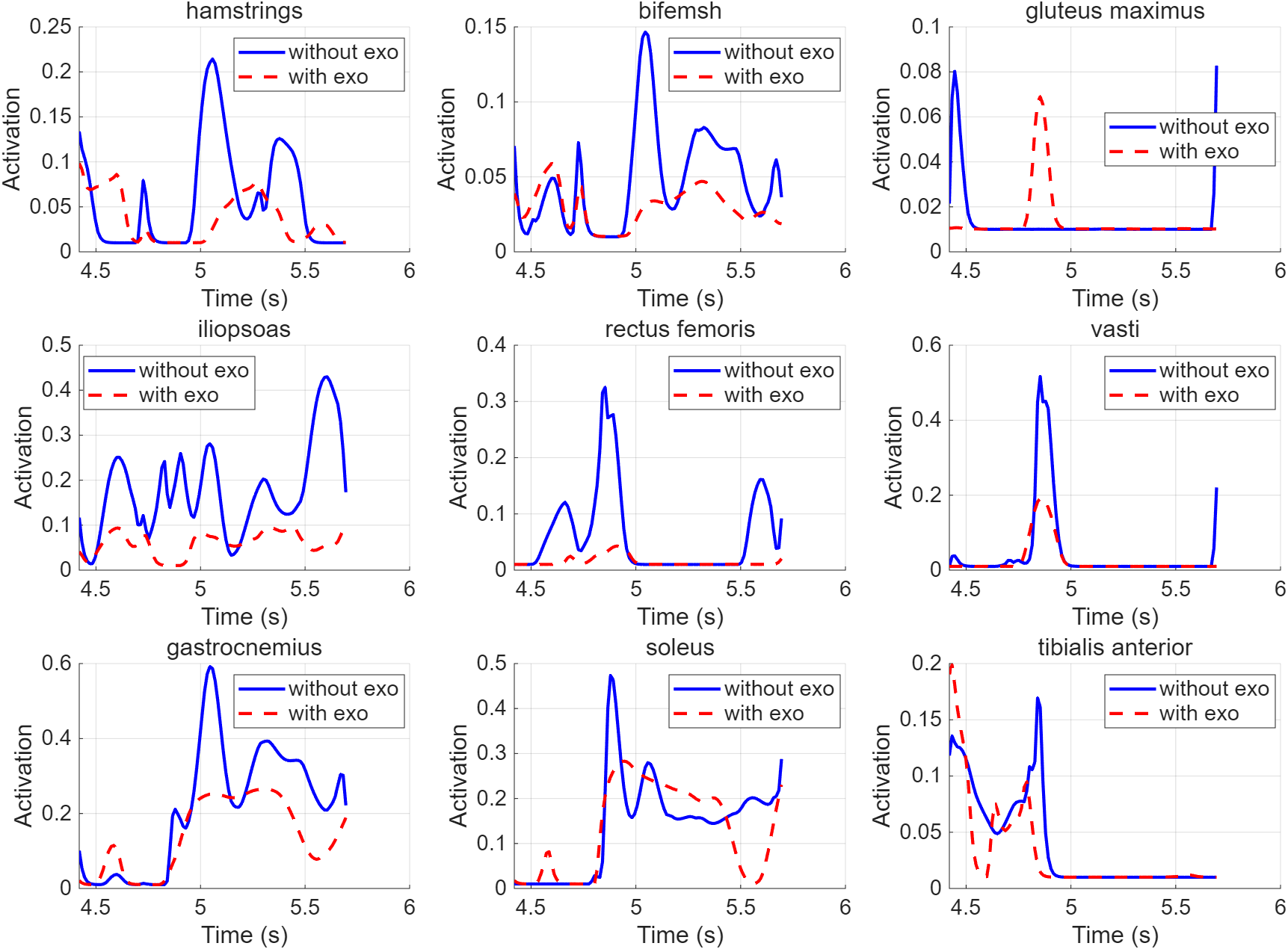


Figure S2: Comparison of right-leg muscle activations during one gait cycle under two conditions: without exoskeleton (blue solid lines) and with knee exoskeleton assistance (red dashed lines). The results illustrated reduced activation levels across major lower-limb muscles when exoskeleton torque support was applied.


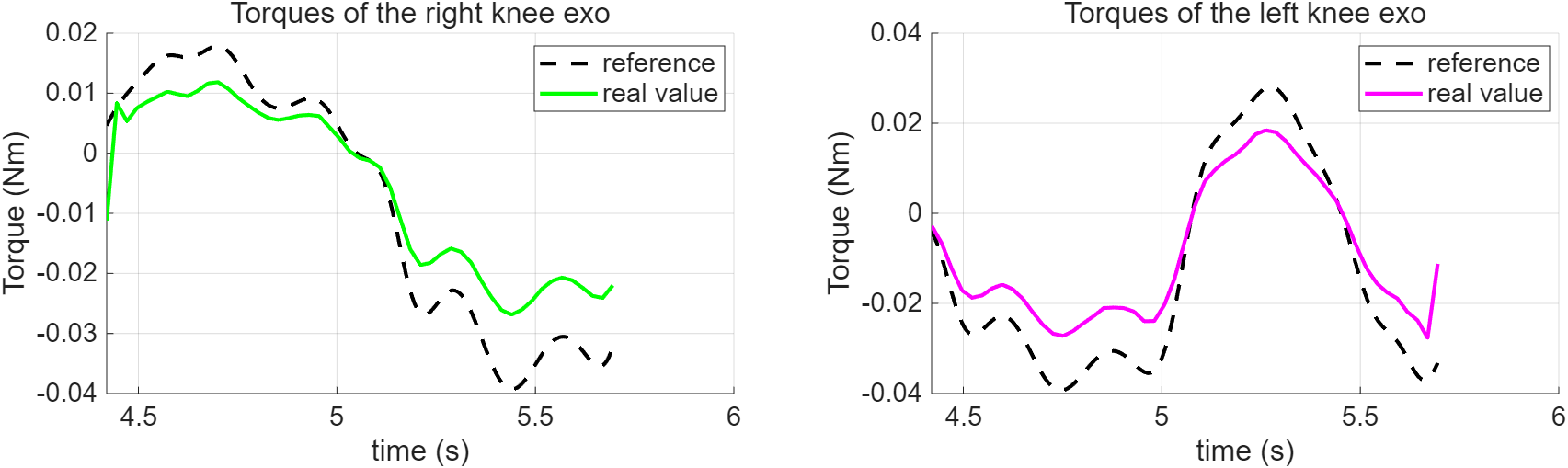


Figure S3: Reference and realized knee exoskeleton torques during one gait cycle. The dashed lines represented the reference torques (20% of the predicted knee joint moments from Marker-GMformer), while the solid lines showed the realized torques applied by the exoskeleton in simulation for the right (green) and left (red) knees.


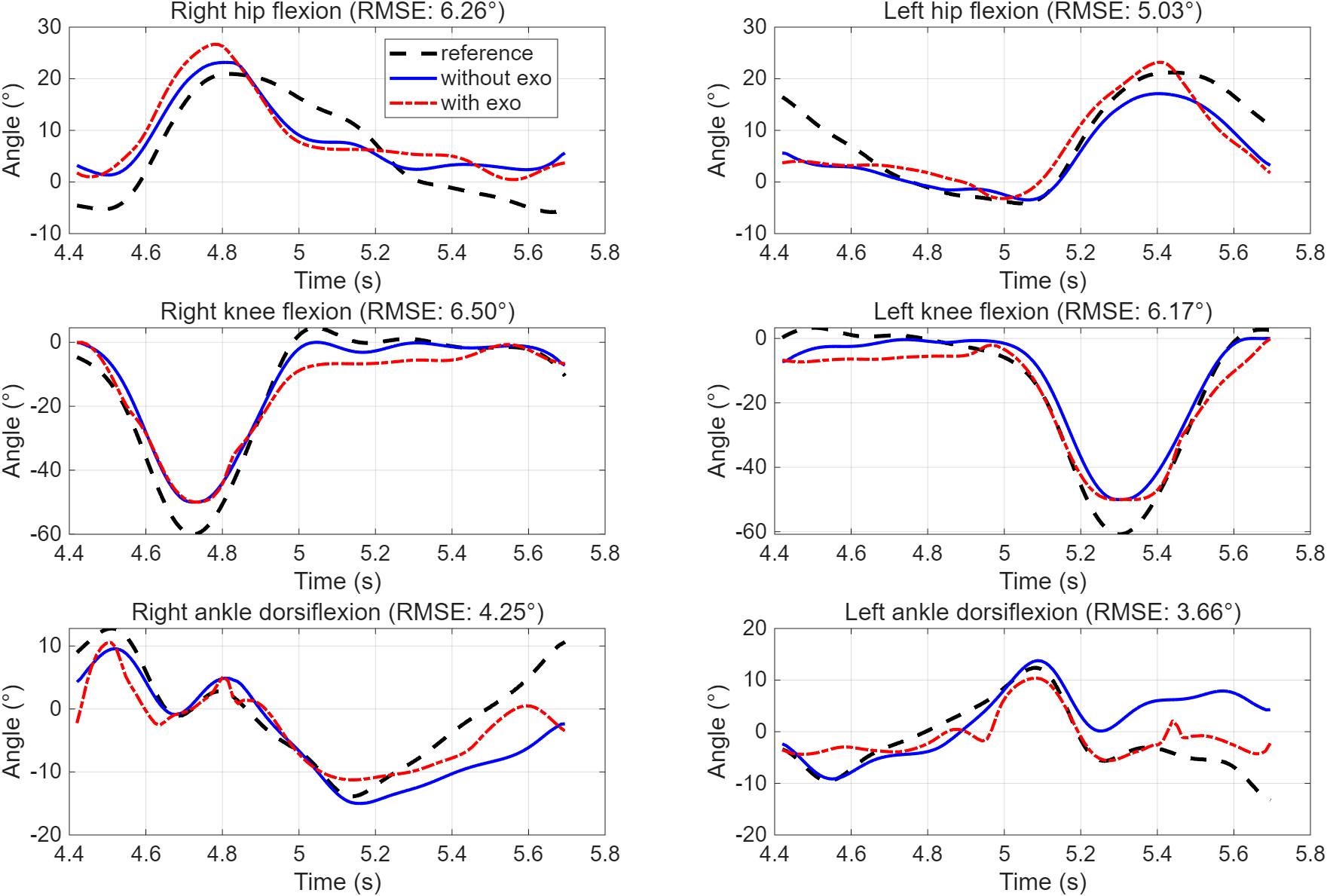


Figure S4: Comparison of lower-limb joint kinematics during one gait cycle under three conditions: experimental reference (black dashed lines), simulated gait without exoskeleton (blue solid lines), and simulated gait with knee exoskeleton assistance (red dash-dotted lines). The results showed that exoskeleton assistance maintained overall kinematic consistency with reference gait while reducing deviation in joint angles across hip, knee, and ankle joints (RMSE values shown in each panel).


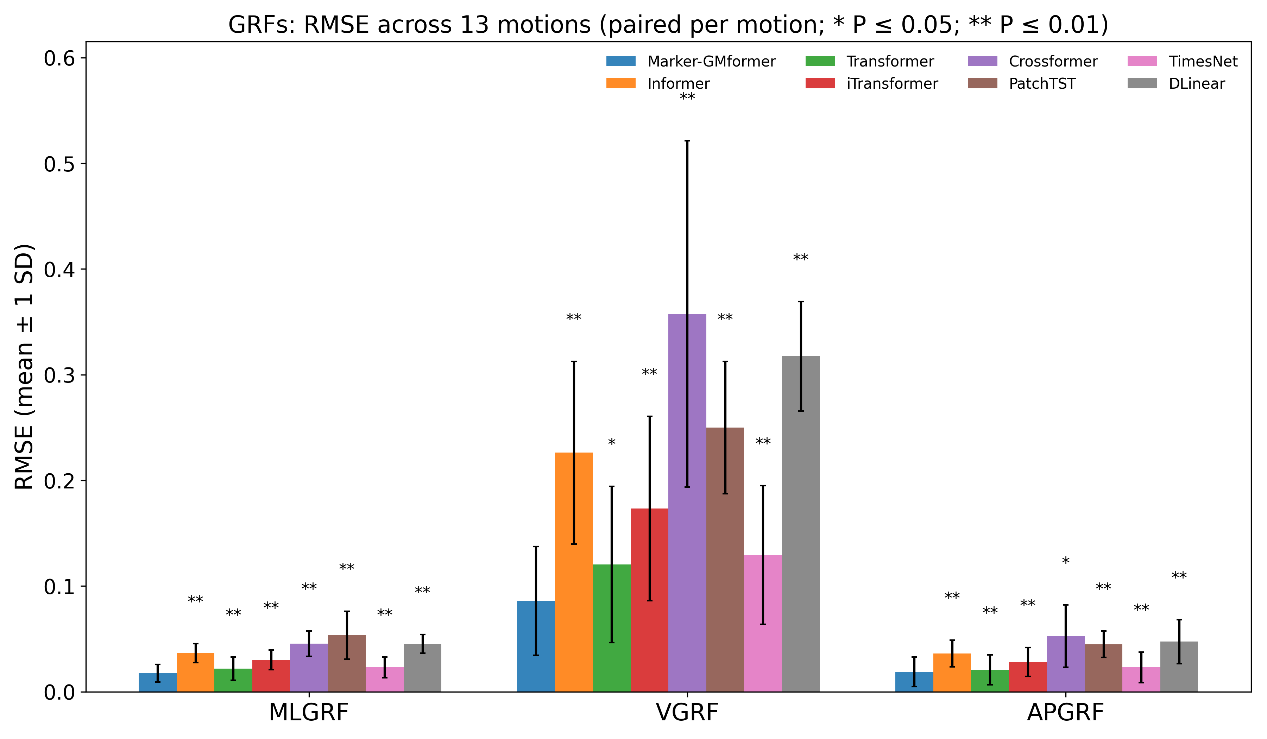


Figure S5: GRFs—model-wise RMSE across 13 motion patterns. Mean ± 1 SD RMSE of MLGRF, VGRF, and APGRF for 8 models, aggregated at the motion level (n = 13). Asterisks above non-Marker-GMformer bars indicate paired t-tests versus Marker-GMformer across motions (*P ≤ 0.05; **P ≤ 0.01).


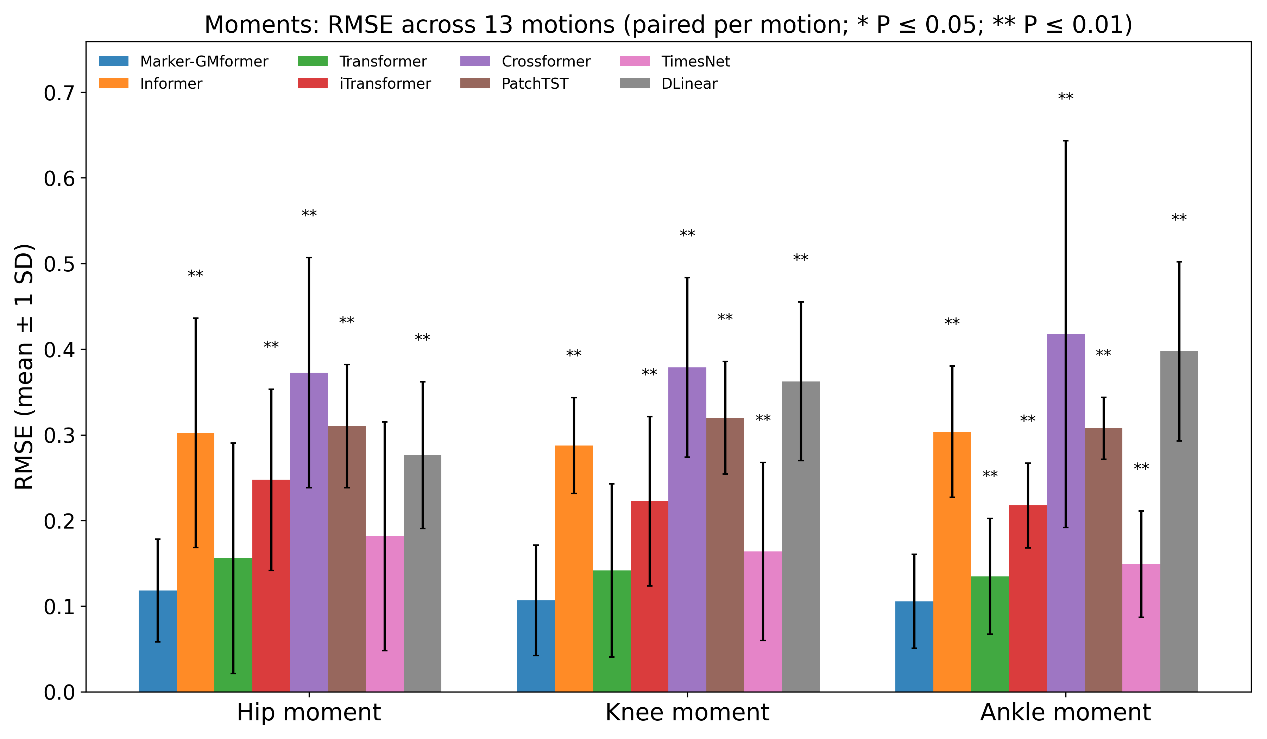


Figure S6: Joint moments—model-wise RMSE across 13 motion patterns. Mean ± 1 SD RMSE of hip, knee, and ankle moments for the 8 models, aggregated at the motion level (n = 13). Significance markers denote paired t-tests versus Marker-GMformer (*P ≤ 0.05; **P ≤ 0.01).


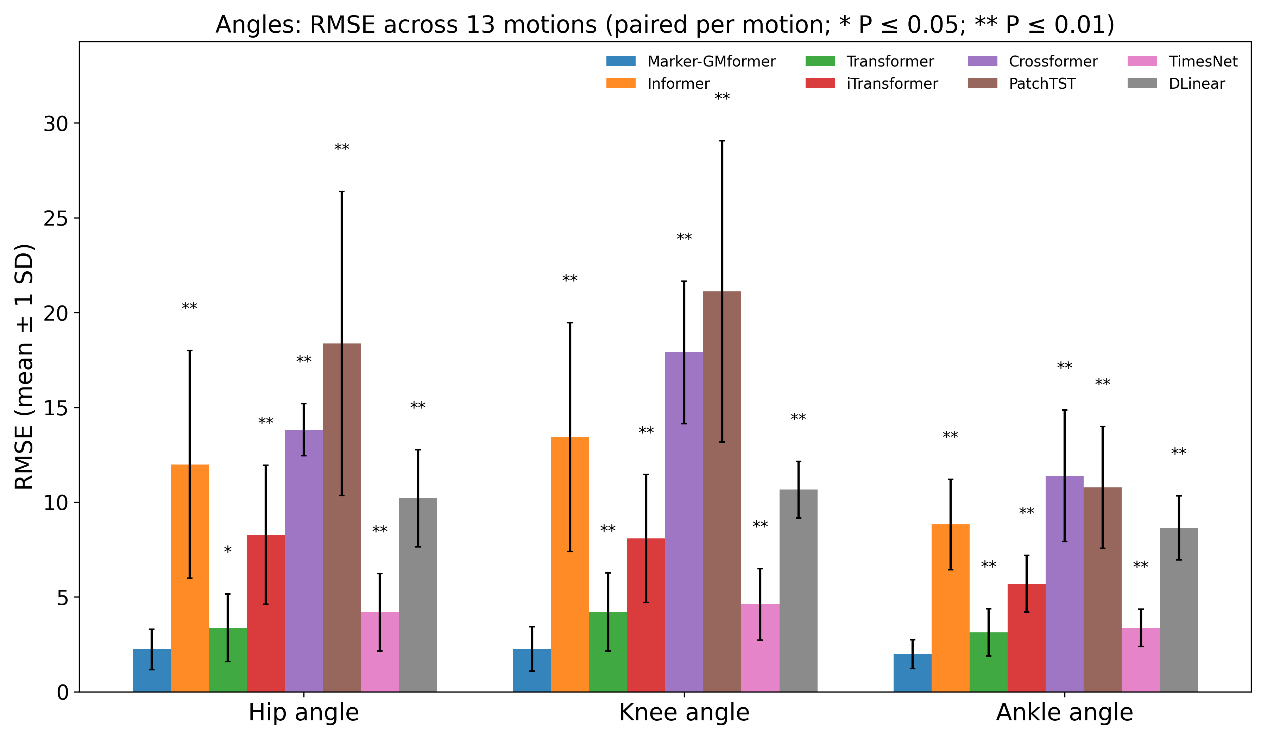


Figure S7. Joint angles—model-wise RMSE across 13 motions patterns. Mean ± 1 SD RMSE of hip, knee, and ankle angles for the 8 models, aggregated at the motion level (n = 13). Asterisks indicate paired t-tests versus Marker-GMformer (*P ≤ 0.05; **P ≤ 0.01).

Table S1: Prediction results of lower limb multi-joint angles, moments, and three-dimensional ground reaction forces (GRFs) during walking tasks across different models (lookback length *T* = 48 time steps).

| Models | Metrics | GRFs | | | Joint Moments | | | Joint Angles | | |
| --- | --- | --- | --- | --- | --- | --- | --- | --- | --- | --- |
|  |  | MLGRF | VGRF | APGRF | Hip | Knee | Ankle | Hip | Knee | Ankle |
| Marker-GMformer | MAE | **0.0056** | **0.021** | **0.006** | **0.037** | **0.027** | **0.033** | **0.73** | **0.8** | **0.68** |
|  | RMSE | **0.0085** | **0.03** | **0.0094** | **0.049** | **0.037** | **0.05** | **0.93** | **1.00** | **0.92** |
|  | *ρ* | **0.99** | **0.998** | **0.996** | **0.99** | **0.99** | **0.996** | **0.998** | **0.999** | **0.99** |
| Informer | MAE | 0.019 | 0.077 | 0.022 | 0.16 | 0.16 | 0.12 | 4.55 | 5.61 | 4.3 |
|  | RMSE | 0.027 | 0.11 | 0.033 | 0.22 | 0.23 | 0.18 | 6.41 | 7.75 | 5.65 |
|  | *ρ* | 0.9 | 0.97 | 0.96 | 0.9 | 0.82 | 0.95 | 0.91 | 0.95 | 0.85 |
| Transformer | MAE | 0.0067 | 0.032 | 0.0072 | 0.041 | 0.035 | 0.044 | 1.08 | 1.66 | 1.08 |
|  | RMSE | 0.01 | 0.042 | 0.012 | 0.054 | 0.049 | 0.064 | 1.37 | 2.05 | 1.45 |
|  | *ρ* | 0.99 | 0.997 | 0.99 | 0.99 | 0.99 | 0.99 | 0.997 | 0.997 | 0.99 |
| iTransformer | MAE | 0.02 | 0.052 | 0.015 | 0.11 | 0.092 | 0.099 | 2.77 | 3.71 | 2.22 |
|  | RMSE | 0.028 | 0.074 | 0.022 | 0.14 | 0.12 | 0.14 | 3.45 | 4.7 | 2.85 |
|  | *ρ* | 0.93 | 0.99 | 0.98 | 0.96 | 0.91 | 0.97 | 0.98 | 0.98 | 0.95 |
| Crossformer | MAE | 0.04 | 0.32 | 0.056 | 0.26 | 0.22 | 0.33 | 10.2 | 14.4 | 5.41 |
|  | RMSE | 0.049 | 0.35 | 0.091 | 0.36 | 0.29 | 0.44 | 12.13 | 17.58 | 7.07 |
|  | *ρ* | 0.78 | 0.84 | 0.74 | 0.8 | 0.23 | 0.71 | 0.65 | 0.56 | 0.57 |
| PatchTST | MAE | 0.026 | 0.14 | 0.029 | 0.18 | 0.17 | 0.18 | 6.83 | 9.48 | 5.08 |
|  | RMSE | 0.033 | 0.18 | 0.042 | 0.23 | 0.22 | 0.24 | 8.68 | 11.88 | 6.48 |
|  | *ρ* | 0.85 | 0.92 | 0.92 | 0.86 | 0.72 | 0.9 | 0.88 | 0.84 | 0.69 |
| TimesNet | MAE | 0.009 | 0.037 | 0.01 | 0.056 | 0.049 | 0.054 | 1.52 | 1.81 | 1.38 |
|  | RMSE | 0.013 | 0.054 | 0.015 | 0.073 | 0.064 | 0.077 | 1.91 | 2.29 | 1.86 |
|  | *ρ* | 0.98 | 0.99 | 0.99 | 0.99 | 0.98 | 0.99 | 0.99 | 0.99 | 0.98 |
| DLinear | MAE | 0.034 | 0.21 | 0.036 | 0.16 | 0.15 | 0.19 | 6.12 | 7.35 | 5.55 |
|  | RMSE | 0.043 | 0.26 | 0.055 | 0.2 | 0.2 | 0.27 | 7.63 | 8.93 | 6.95 |
|  | *ρ* | 0.76 | 0.82 | 0.88 | 0.9 | 0.76 | 0.87 | 0.87 | 0.9 | 0.67 |

Table S2: Prediction results of lower limb multi-joint angles, moments, and three-dimensional ground reaction forces (GRFs) during inclined walking tasks across different models (lookback length *T* = 48 time steps).

| Models | Metrics | GRFs | | | Joint Moments | | | Joint Angles | | |
| --- | --- | --- | --- | --- | --- | --- | --- | --- | --- | --- |
|  |  | MLGRF | VGRF | APGRF | Hip | Knee | Ankle | Hip | Knee | Ankle |
| Marker-GMformer | MAE | **0.0065** | **0.024** | **0.0071** | **0.048** | **0.037** | **0.04** | **0.9** | **0.96** | **0.75** |
|  | RMSE | **0.0096** | **0.035** | **0.011** | **0.065** | **0.054** | **0.062** | **1.18** | **1.29** | **1.01** |
|  | *ρ* | **0.99** | **0.997** | **0.99** | **0.99** | **0.99** | **0.99** | **0.998** | **0.998** | **0.996** |
| Informer | MAE | 0.018 | 0.075 | 0.024 | 0.16 | 0.18 | 0.14 | 6.63 | 5.59 | 5.04 |
|  | RMSE | 0.024 | 0.1 | 0.036 | 0.2 | 0.26 | 0.23 | 8.23 | 7.68 | 6.47 |
|  | *ρ* | 0.93 | 0.98 | 0.95 | 0.93 | 0.87 | 0.93 | 0.93 | 0.93 | 0.88 |
| Transformer | MAE | 0.0074 | 0.033 | 0.0082 | 0.055 | 0.047 | 0.052 | 1.41 | 1.71 | 1.22 |
|  | RMSE | 0.011 | 0.046 | 0.013 | 0.073 | 0.069 | 0.079 | 1.78 | 2.14 | 1.68 |
|  | *ρ* | 0.99 | 0.996 | 0.99 | 0.99 | 0.98 | 0.99 | 0.997 | 0.996 | 0.99 |
| iTransformer | MAE | 0.019 | 0.059 | 0.017 | 0.13 | 0.11 | 0.13 | 3.61 | 3.69 | 3.38 |
|  | RMSE | 0.025 | 0.083 | 0.024 | 0.17 | 0.15 | 0.19 | 4.48 | 4.69 | 4.33 |
|  | *ρ* | 0.95 | 0.99 | 0.97 | 0.94 | 0.92 | 0.95 | 0.99 | 0.97 | 0.95 |
| Crossformer | MAE | 0.04 | 0.3 | 0.052 | 0.28 | 0.27 | 0.34 | 12.94 | 14.44 | 7.72 |
|  | RMSE | 0.045 | 0.34 | 0.073 | 0.35 | 0.36 | 0.45 | 14.89 | 17.26 | 9.55 |
|  | *ρ* | 0.84 | 0.87 | 0.75 | 0.88 | 0.26 | 0.79 | 0.83 | 0.6 | 0.67 |
| PatchTST | MAE | 0.021 | 0.12 | 0.026 | 0.2 | 0.18 | 0.21 | 7.2 | 9.36 | 6.17 |
|  | RMSE | 0.027 | 0.15 | 0.034 | 0.25 | 0.25 | 0.28 | 9.25 | 12.08 | 7.61 |
|  | *ρ* | 0.9 | 0.94 | 0.93 | 0.85 | 0.74 | 0.88 | 0.9 | 0.81 | 0.79 |
| TimesNet | MAE | 0.0091 | 0.038 | 0.01 | 0.07 | 0.062 | 0.067 | 1.71 | 2.19 | 1.66 |
|  | RMSE | 0.013 | 0.055 | 0.015 | 0.095 | 0.087 | 0.097 | 2.18 | 2.86 | 2.18 |
|  | *ρ* | 0.97 | 0.99 | 0.99 | 0.98 | 0.97 | 0.99 | 0.99 | 0.99 | 0.98 |
| DLinear | MAE | 0.028 | 0.19 | 0.036 | 0.18 | 0.19 | 0.23 | 6.91 | 7.72 | 6.02 |
|  | RMSE | 0.035 | 0.24 | 0.049 | 0.23 | 0.26 | 0.31 | 8.56 | 9.42 | 7.5 |
|  | *ρ* | 0.83 | 0.85 | 0.85 | 0.88 | 0.73 | 0.84 | 0.92 | 0.9 | 0.81 |

Table S3: Prediction results of lower limb multi-joint angles, moments, and three-dimensional ground reaction forces (GRFs) during running tasks across different models (lookback length *T* = 48 time steps).

| Models | Metrics | GRFs | | | Joint Moments | | | Joint Angles | | |
| --- | --- | --- | --- | --- | --- | --- | --- | --- | --- | --- |
|  |  | MLGRF | VGRF | APGRF | Hip | Knee | Ankle | Hip | Knee | Ankle |
| Marker-GMformer | MAE | **0.0071** | **0.031** | **0.0066** | **0.054** | **0.034** | **0.034** | **0.94** | **0.92** | **1.1** |
|  | RMSE | **0.014** | **0.053** | **0.011** | **0.072** | **0.048** | **0.053** | **1.19** | **1.18** | **1.84** |
|  | *ρ* | **0.98** | **0.998** | **0.99** | **0.995** | **0.997** | **0.999** | **0.997** | **0.999** | **0.99** |
| Informer | MAE | 0.021 | 0.13 | 0.022 | 0.19 | 0.15 | 0.2 | 5.02 | 6.6 | 4.81 |
|  | RMSE | 0.04 | 0.21 | 0.036 | 0.24 | 0.21 | 0.36 | 6.28 | 8.02 | 7.17 |
|  | *ρ* | 0.79 | 0.97 | 0.92 | 0.95 | 0.95 | 0.97 | 0.92 | 0.96 | 0.92 |
| Transformer | MAE | 0.008 | 0.038 | 0.0075 | 0.061 | 0.045 | 0.048 | 1.31 | 1.83 | 1.58 |
|  | RMSE | 0.016 | 0.061 | 0.014 | 0.081 | 0.066 | 0.078 | 1.68 | 2.29 | 2.41 |
|  | *ρ* | 0.97 | 0.998 | 0.99 | 0.99 | 0.996 | 0.998 | 0.995 | 0.998 | 0.99 |
| iTransformer | MAE | 0.024 | 0.099 | 0.019 | 0.24 | 0.13 | 0.17 | 4.39 | 3.5 | 4.14 |
|  | RMSE | 0.033 | 0.15 | 0.027 | 0.3 | 0.16 | 0.28 | 5.69 | 4.55 | 5.52 |
|  | *ρ* | 0.91 | 0.99 | 0.96 | 0.97 | 0.97 | 0.97 | 0.96 | 0.99 | 0.95 |
| Crossformer | MAE | 0.036 | 0.61 | 0.049 | 0.56 | 0.44 | 0.72 | 13.02 | 20.5 | 12.16 |
|  | RMSE | 0.061 | 0.72 | 0.086 | 0.67 | 0.62 | 0.91 | 15.07 | 25.26 | 14.93 |
|  | *ρ* | 0.37 | 0.7 | 0.42 | 0.66 | 0.39 | 0.43 | 0.15 | 0.34 | 0.48 |
| PatchTST | MAE | 0.035 | 0.18 | 0.037 | 0.27 | 0.22 | 0.24 | 7.92 | 10.25 | 7.98 |
|  | RMSE | 0.053 | 0.24 | 0.049 | 0.36 | 0.29 | 0.34 | 10.57 | 13.53 | 10.22 |
|  | *ρ* | 0.6 | 0.95 | 0.84 | 0.88 | 0.9 | 0.94 | 0.8 | 0.87 | 0.82 |
| TimesNet | MAE | 0.0099 | 0.045 | 0.011 | 0.072 | 0.058 | 0.052 | 1.7 | 1.88 | 2.15 |
|  | RMSE | 0.018 | 0.077 | 0.018 | 0.093 | 0.08 | 0.079 | 2.16 | 2.37 | 3.08 |
|  | *ρ* | 0.96 | 0.99 | 0.98 | 0.99 | 0.99 | 0.997 | 0.99 | 0.996 | 0.98 |
| DLinear | MAE | 0.043 | 0.26 | 0.068 | 0.22 | 0.39 | 0.39 | 5.76 | 7.24 | 8.83 |
|  | RMSE | 0.057 | 0.35 | 0.082 | 0.28 | 0.48 | 0.58 | 7.16 | 9.07 | 11.22 |
|  | *ρ* | 0.62 | 0.9 | 0.53 | 0.93 | 0.71 | 0.85 | 0.89 | 0.95 | 0.74 |

Table S4: Prediction results of lower limb multi-joint angles, moments, and three-dimensional ground reaction forces (GRFs) during squatting task across different models (lookback length *T* = 48 time steps).

| Models | Metrics | GRFs | | | Joint Moments | | | Joint Angles | | |
| --- | --- | --- | --- | --- | --- | --- | --- | --- | --- | --- |
|  |  | MLGRF | VGRF | APGRF | Hip | Knee | Ankle | Hip | Knee | Ankle |
| Marker-GMformer | MAE | **0.02** | **0.086** | **0.0093** | **0.15** | **0.15** | **0.081** | **2.39** | **2.75** | **1.77** |
|  | RMSE | **0.029** | **0.11** | **0.013** | **0.23** | **0.22** | **0.11** | **3.47** | **4.54** | **2.39** |
|  | *ρ* | **0.89** | **0.84** | 0.39 | **0.6** | **0.91** | **0.85** | **0.997** | **0.995** | **0.99** |
| Informer | MAE | 0.036 | 0.2 | 0.016 | 0.42 | 0.22 | 0.24 | 19.3 | 19.05 | 9.79 |
|  | RMSE | 0.044 | 0.24 | 0.021 | 0.59 | 0.3 | 0.32 | 24.2 | 23.19 | 11.01 |
|  | *ρ* | 0.78 | 0.15 | 0.11 | 0.48 | 0.71 | 0.043 | 0.9 | 0.93 | 0.88 |
| Transformer | MAE | 0.023 | 0.16 | 0.0098 | 0.28 | 0.25 | 0.11 | 4 | 5.02 | 2.84 |
|  | RMSE | 0.036 | 0.22 | 0.013 | 0.47 | 0.36 | 0.17 | 5.4 | 7.47 | 4.28 |
|  | *ρ* | 0.84 | 0.4 | 0.43 | 0.54 | 0.84 | 0.7 | 0.99 | 0.99 | 0.97 |
| iTransformer | MAE | 0.03 | 0.29 | 0.012 | 0.34 | 0.36 | 0.14 | 11.63 | 11.41 | 5.02 |
|  | RMSE | 0.036 | 0.34 | 0.015 | 0.46 | 0.44 | 0.19 | 14.03 | 14.25 | 6.26 |
|  | *ρ* | 0.78 | 0.2 | 0.077 | 0.48 | 0.77 | 0.48 | 0.96 | 0.98 | 0.94 |
| Crossformer | MAE | 0.031 | 0.17 | 0.011 | 0.33 | 0.31 | 0.15 | 13.17 | 12.75 | 6.87 |
|  | RMSE | 0.04 | 0.21 | 0.014 | 0.42 | 0.35 | 0.18 | 15.72 | 14.84 | 7.72 |
|  | *ρ* | 0.8 | 0.39 | 0.11 | 0.45 | 0.83 | 0.49 | 0.98 | 0.99 | 0.97 |
| PatchTST | MAE | 0.054 | 0.29 | 0.021 | 0.23 | 0.28 | 0.26 | 24.13 | 19.94 | 6.66 |
|  | RMSE | 0.063 | 0.35 | 0.027 | 0.3 | 0.35 | 0.31 | 28.03 | 23.82 | 8.25 |
|  | *ρ* | -0.022 | -0.39 | 0.26 | 0.36 | 0.78 | 0.033 | 0.85 | 0.86 | 0.87 |
| TimesNet | MAE | 0.024 | 0.15 | 0.011 | 0.29 | 0.24 | 0.12 | 3.9 | 4.25 | 2.27 |
|  | RMSE | 0.037 | 0.22 | 0.014 | 0.48 | 0.38 | 0.18 | 4.9 | 6.27 | 3.32 |
|  | *ρ* | 0.83 | 0.42 | 0.34 | 0.5 | 0.81 | 0.69 | 0.99 | 0.99 | 0.98 |
| DLinear | MAE | 0.029 | 0.28 | 0.013 | 0.31 | 0.34 | 0.32 | 10.96 | 9.45 | 5.3 |
|  | RMSE | 0.036 | 0.37 | 0.018 | 0.43 | 0.39 | 0.4 | 13.28 | 12.05 | 6.61 |
|  | *ρ* | 0.78 | -0.33 | **0.053** | 0.52 | 0.79 | -0.31 | 0.97 | 0.97 | 0.93 |

Table S5: Prediction results of lower limb multi-joint angles, moments, and three-dimensional ground reaction forces (GRFs) during stairs ascent and descent tasks across different models (lookback length *T* = 48 time steps).

| Models | Metrics | GRFs | | | Joint Moments | | | Joint Angles | | |
| --- | --- | --- | --- | --- | --- | --- | --- | --- | --- | --- |
|  |  | MLGRF | VGRF | APGRF | Hip | Knee | Ankle | Hip | Knee | Ankle |
| Marker-GMformer | MAE | **0.0076** | **0.055** | **0.011** | **0.087** | **0.085** | **0.08** | **1.74** | **1.75** | **1.54** |
|  | RMSE | **0.011** | **0.08** | **0.016** | **0.12** | **0.13** | **0.12** | **2.19** | **2.25** | **2.03** |
|  | *ρ* | **0.97** | **0.98** | **0.92** | **0.93** | **0.96** | **0.96** | **0.99** | **0.997** | **0.99** |
| Informer | MAE | 0.019 | 0.22 | 0.018 | 0.21 | 0.24 | 0.22 | 7.36 | 7.52 | 10.35 |
|  | RMSE | 0.029 | 0.33 | 0.028 | 0.27 | 0.35 | 0.34 | 8.98 | 9.33 | 12.12 |
|  | *ρ* | 0.8 | 0.85 | 0.73 | 0.6 | 0.71 | 0.73 | 0.89 | 0.95 | 0.91 |
| Transformer | MAE | 0.0089 | 0.066 | 0.012 | 0.095 | 0.092 | 0.094 | 2.87 | 3.14 | 2.8 |
|  | RMSE | 0.014 | 0.11 | 0.019 | 0.13 | 0.14 | 0.15 | 3.54 | 4.13 | 3.59 |
|  | *ρ* | 0.96 | 0.98 | 0.9 | 0.92 | 0.95 | 0.95 | 0.98 | 0.99 | 0.98 |
| iTransformer | MAE | 0.014 | 0.11 | 0.016 | 0.18 | 0.15 | 0.18 | 5.94 | 6.43 | 4.6 |
|  | RMSE | 0.019 | 0.15 | 0.022 | 0.23 | 0.2 | 0.24 | 7.52 | 8.16 | 5.96 |
|  | *ρ* | 0.91 | 0.95 | 0.85 | 0.77 | 0.88 | 0.83 | 0.92 | 0.96 | 0.93 |
| Crossformer | MAE | 0.026 | 0.34 | 0.025 | 0.26 | 0.32 | 0.32 | 11.95 | 18.78 | 10.87 |
|  | RMSE | 0.033 | 0.38 | 0.035 | 0.31 | 0.38 | 0.38 | 14 | 21.84 | 14.82 |
|  | *ρ* | 0.71 | 0.83 | 0.54 | 0.54 | 0.61 | 0.65 | 0.77 | 0.79 | 0.7 |
| PatchTST | MAE | 0.038 | 0.19 | 0.037 | 0.27 | 0.31 | 0.25 | 16.07 | 22.86 | 11.57 |
|  | RMSE | 0.048 | 0.25 | 0.048 | 0.34 | 0.41 | 0.32 | 20.02 | 28.01 | 14.61 |
|  | *ρ* | 0.21 | 0.84 | 0.18 | 0.38 | 0.47 | 0.73 | 0.32 | 0.28 | 0.41 |
| TimesNet | MAE | 0.011 | 0.093 | 0.014 | 0.16 | 0.13 | 0.13 | 4.15 | 4.53 | 3.29 |
|  | RMSE | 0.017 | 0.14 | 0.02 | 0.2 | 0.18 | 0.18 | 5.2 | 5.93 | 4.34 |
|  | *ρ* | 0.92 | 0.96 | 0.87 | 0.79 | 0.91 | 0.92 | 0.96 | 0.98 | 0.96 |
| DLinear | MAE | 0.036 | 0.31 | 0.032 | 0.23 | 0.31 | 0.39 | 10.9 | 10.65 | 7.81 |
|  | RMSE | 0.044 | 0.39 | 0.04 | 0.29 | 0.4 | 0.52 | 13.38 | 12.86 | 10.04 |
|  | *ρ* | 0.58 | 0.59 | 0.54 | 0.5 | 0.38 | 0.28 | 0.66 | 0.89 | 0.78 |

Table S6: Prediction results of lower-limb joint angles, joint moments, and three-dimensional ground reaction forces (GRFs) during step-up tasks across different models (lookback length *T* = 48 time steps).

| Models | Metrics | GRFs | | | Joint Moments | | | Joint Angles | | |
| --- | --- | --- | --- | --- | --- | --- | --- | --- | --- | --- |
|  |  | MLGRF | VGRF | APGRF | Hip | Knee | Ankle | Hip | Knee | Ankle |
| Marker-GMformer | MAE | **0.012** | **0.046** | **0.011** | **0.075** | **0.062** | **0.064** | **1.71** | **1.8** | **1.43** |
|  | RMSE | **0.016** | **0.07** | **0.016** | **0.1** | **0.085** | **0.09** | **2.22** | **2.29** | **1.85** |
|  | *ρ* | **0.91** | **0.98** | **0.9** | **0.97** | **0.98** | **0.94** | **0.998** | **0.999** | **0.99** |
| Informer | MAE | 0.028 | 0.24 | 0.025 | 0.26 | 0.24 | 0.17 | 12.23 | 15.73 | 7.99 |
|  | RMSE | 0.038 | 0.33 | 0.035 | 0.32 | 0.36 | 0.25 | 15.74 | 18.73 | 9.82 |
|  | *ρ* | 0.58 | 0.64 | 0.54 | 0.8 | 0.66 | 0.46 | 0.94 | 0.96 | 0.72 |
| Transformer | MAE | 0.014 | 0.062 | 0.011 | 0.087 | 0.078 | 0.066 | 2.57 | 3.08 | 2.29 |
|  | RMSE | 0.021 | 0.095 | 0.016 | 0.12 | 0.11 | 0.098 | 3.18 | 3.89 | 3.01 |
|  | *ρ* | 0.88 | 0.98 | 0.89 | 0.96 | 0.98 | 0.93 | 0.997 | 0.998 | 0.98 |
| iTransformer | MAE | 0.016 | 0.11 | 0.018 | 0.2 | 0.17 | 0.13 | 8.15 | 6.72 | 5.02 |
|  | RMSE | 0.023 | 0.14 | 0.025 | 0.27 | 0.23 | 0.18 | 9.94 | 8.92 | 6.26 |
|  | *ρ* | 0.81 | 0.94 | 0.79 | 0.89 | 0.86 | 0.75 | 0.97 | 0.98 | 0.88 |
| Crossformer | MAE | 0.019 | 0.15 | 0.021 | 0.25 | 0.22 | 0.14 | 10.21 | 13.13 | 6.92 |
|  | RMSE | 0.026 | 0.18 | 0.028 | 0.31 | 0.29 | 0.19 | 12.13 | 15.73 | 8.54 |
|  | *ρ* | 0.75 | 0.91 | 0.62 | 0.76 | 0.79 | 0.69 | 0.97 | 0.96 | 0.85 |
| PatchTST | MAE | 0.028 | 0.22 | 0.036 | 0.29 | 0.26 | 0.22 | 22.69 | 27.11 | 9.71 |
|  | RMSE | 0.035 | 0.27 | 0.045 | 0.36 | 0.34 | 0.29 | 28.06 | 33.57 | 12.61 |
|  | *ρ* | 0.7 | 0.73 | 0.42 | 0.58 | 0.67 | 0.49 | 0.7 | 0.74 | 0.38 |
| TimesNet | MAE | 0.015 | 0.076 | 0.013 | 0.11 | 0.096 | 0.093 | 3.11 | 3.58 | 2.65 |
|  | RMSE | 0.022 | 0.11 | 0.019 | 0.15 | 0.13 | 0.13 | 3.93 | 4.74 | 3.6 |
|  | *ρ* | 0.85 | 0.96 | 0.85 | 0.94 | 0.96 | 0.87 | 0.99 | 0.99 | 0.96 |
| DLinear | MAE | 0.03 | 0.25 | 0.025 | 0.23 | 0.35 | 0.26 | 6.71 | 8.06 | 7.05 |
|  | RMSE | 0.039 | 0.32 | 0.033 | 0.3 | 0.44 | 0.37 | 8.59 | 9.97 | 8.81 |
|  | *ρ* | 0.37 | 0.66 | 0.47 | 0.77 | 0.36 | 0.31 | 0.97 | 0.98 | 0.75 |

Table S7: Prediction results of lower-limb joint angles, joint moments, and three-dimensional ground reaction forces (GRFs) during vertical jump task across different models (lookback length *T* = 48 time steps).

| Models | Metrics | GRFs | | | Joint Moments | | | Joint Angles | | |
| --- | --- | --- | --- | --- | --- | --- | --- | --- | --- | --- |
|  |  | MLGRF | VGRF | APGRF | Hip | Knee | Ankle | Hip | Knee | Ankle |
| Marker-GMformer | MAE | **0.019** | **0.082** | **0.022** | **0.11** | **0.099** | **0.1** | **2.43** | **2.14** | **1.8** |
|  | RMSE | **0.026** | **0.13** | **0.052** | **0.16** | **0.17** | **0.15** | **3.26** | **2.93** | **3.01** |
|  | *ρ* | **0.94** | **0.93** | **0.61** | **0.95** | **0.95** | **0.94** | **0.99** | **0.996** | **0.99** |
| Informer | MAE | 0.04 | 0.19 | 0.033 | 0.28 | 0.24 | 0.33 | 10.9 | 15.13 | 8.78 |
|  | RMSE | 0.05 | 0.25 | 0.065 | 0.39 | 0.33 | 0.41 | 13.1 | 17.63 | 10.68 |
|  | *ρ* | 0.78 | 0.76 | 0.24 | 0.81 | 0.81 | 0.69 | 0.9 | 0.91 | 0.95 |
| Transformer | MAE | 0.022 | 0.11 | 0.023 | 0.14 | 0.13 | 0.12 | 4.23 | 4.61 | 3.11 |
|  | RMSE | 0.033 | 0.17 | 0.054 | 0.2 | 0.2 | 0.18 | 6.27 | 6.45 | 4.98 |
|  | *ρ* | 0.89 | 0.89 | 0.58 | 0.93 | 0.93 | 0.91 | 0.98 | 0.98 | 0.98 |
| iTransformer | MAE | 0.034 | 0.18 | 0.028 | 0.19 | 0.19 | 0.19 | 7.63 | 8.08 | 5.49 |
|  | RMSE | 0.049 | 0.23 | 0.06 | 0.27 | 0.25 | 0.25 | 9.4 | 10.18 | 7.59 |
|  | *ρ* | 0.74 | 0.82 | 0.41 | 0.86 | 0.89 | 0.81 | 0.95 | 0.96 | 0.94 |
| Crossformer | MAE | 0.044 | 0.22 | 0.029 | 0.26 | 0.3 | 0.27 | 10.41 | 13.26 | 10.6 |
|  | RMSE | 0.056 | 0.31 | 0.065 | 0.35 | 0.4 | 0.36 | 12.7 | 16.23 | 14.5 |
|  | *ρ* | 0.64 | 0.61 | 0.036 | 0.78 | 0.7 | 0.6 | 0.92 | 0.92 | 0.85 |
| PatchTST | MAE | 0.071 | 0.21 | 0.038 | 0.29 | 0.28 | 0.25 | 15.43 | 19.08 | 11.38 |
|  | RMSE | 0.089 | 0.29 | 0.07 | 0.42 | 0.39 | 0.35 | 19.26 | 22.77 | 15.1 |
|  | *ρ* | 0.096 | 0.58 | 0.11 | 0.6 | 0.69 | 0.6 | 0.75 | 0.81 | 0.82 |
| TimesNet | MAE | 0.026 | 0.14 | 0.026 | 0.16 | 0.15 | 0.16 | 5.4 | 5.21 | 3.23 |
|  | RMSE | 0.037 | 0.19 | 0.058 | 0.24 | 0.23 | 0.24 | 7.72 | 7.12 | 4.7 |
|  | *ρ* | 0.86 | 0.84 | 0.46 | 0.89 | 0.91 | 0.85 | 0.97 | 0.98 | 0.98 |
| DLinear | MAE | 0.042 | 0.22 | 0.035 | 0.23 | 0.27 | 0.25 | 8.71 | 9.65 | 7.69 |
|  | RMSE | 0.055 | 0.3 | 0.069 | 0.33 | 0.39 | 0.34 | 10.51 | 11.6 | 10.2 |
|  | *ρ* | 0.66 | 0.55 | -0.1 | 0.79 | 0.73 | 0.61 | 0.94 | 0.95 | 0.92 |

Table S8: Prediction results of lower-limb joint angles, joint moments, and three-dimensional ground reaction forces (GRFs) during hopping task across different models (lookback length *T* = 48 time steps).

| Models | Metrics | GRFs | | | Joint Moments | | | Joint Angles | | |
| --- | --- | --- | --- | --- | --- | --- | --- | --- | --- | --- |
|  |  | MLGRF | VGRF | APGRF | Hip | Knee | Ankle | Hip | Knee | Ankle |
| Marker-GMformer | MAE | **0.017** | **0.12** | **0.015** | 0.11 | **0.08** | **0.13** | **2.6** | **1.99** | **2.06** |
|  | RMSE | **0.026** | **0.18** | **0.023** | 0.15 | **0.11** | **0.21** | **3.45** | **2.62** | **2.84** |
|  | *ρ* | **0.91** | **0.91** | **0.71** | 0.59 | **0.96** | **0.93** | **0.94** | **0.98** | **0.98** |
| Informer | MAE | 0.033 | 0.19 | 0.026 | 0.15 | 0.19 | 0.28 | 9.44 | 11.75 | 6.21 |
|  | RMSE | 0.041 | 0.24 | 0.036 | 0.19 | 0.26 | 0.34 | 12.95 | 15.13 | 7.67 |
|  | *ρ* | 0.78 | 0.79 | 0.29 | 0.58 | 0.71 | 0.78 | 0.38 | 0.56 | 0.93 |
| Transformer | MAE | 0.023 | 0.14 | 0.015 | **0.08** | 0.09 | 0.16 | 2.72 | 4.14 | 2.69 |
|  | RMSE | 0.035 | 0.22 | 0.025 | **0.12** | 0.14 | 0.26 | 3.82 | 5.28 | 3.69 |
|  | *ρ* | 0.89 | 0.89 | 0.68 | **0.76** | 0.95 | 0.91 | 0.94 | 0.95 | 0.97 |
| iTransformer | MAE | 0.02 | 0.17 | 0.02 | 0.11 | 0.16 | 0.19 | 10.02 | 7.29 | 5.31 |
|  | RMSE | 0.029 | 0.22 | 0.031 | 0.14 | 0.23 | 0.27 | 11.74 | 9.19 | 6.85 |
|  | *ρ* | 0.88 | 0.88 | 0.45 | 0.57 | 0.82 | 0.85 | 0.64 | 0.87 | 0.92 |
| Crossformer | MAE | 0.041 | 0.28 | 0.018 | 0.18 | 0.28 | 0.3 | 11.99 | 12.7 | 10.39 |
|  | RMSE | 0.055 | 0.37 | 0.03 | 0.21 | 0.34 | 0.43 | 13.93 | 14.47 | 13.95 |
|  | *ρ* | 0.12 | 0.31 | 0.28 | 0.34 | 0.48 | 0.48 | 0.6 | 0.68 | 0.44 |
| PatchTST | MAE | 0.064 | 0.2 | 0.034 | 0.17 | 0.23 | 0.24 | 19.03 | 19.32 | 8.75 |
|  | RMSE | 0.081 | 0.27 | 0.045 | 0.22 | 0.31 | 0.33 | 23.08 | 23.26 | 11.35 |
|  | *ρ* | 0.38 | 0.71 | 0.036 | 0.35 | 0.6 | 0.75 | 0.24 | 0.63 | 0.8 |
| TimesNet | MAE | 0.019 | 0.14 | 0.018 | 0.087 | 0.11 | 0.16 | 4.26 | 4.22 | 3.03 |
|  | RMSE | 0.028 | 0.19 | 0.026 | 0.12 | 0.16 | 0.21 | 5.5 | 5.31 | 3.9 |
|  | *ρ* | 0.87 | 0.88 | 0.55 | 0.7 | 0.93 | 0.91 | 0.85 | 0.93 | 0.97 |
| DLinear | MAE | 0.037 | 0.24 | 0.024 | 0.12 | 0.25 | 0.26 | 9.32 | 9.28 | 5.65 |
|  | RMSE | 0.054 | 0.31 | 0.034 | 0.15 | 0.34 | 0.39 | 12.5 | 11.39 | 7.82 |
|  | *ρ* | 0.15 | 0.59 | 0.15 | 0.34 | 0.36 | 0.68 | 0.31 | 0.62 | 0.89 |

Table S9: Detailed ablation study results showing the effect of component removal on all target variables (lookback length T = 48 time steps).

| Spatial Block | | Temporal Block | Metrics | GRFs | | | Joint Moments | | | Joint Angles | | |
| --- | --- | --- | --- | --- | --- | --- | --- | --- | --- | --- | --- | --- |
| JM-GCN | TM-GCN |  |  | MLGRF | VGRF | APGRF | Hip | Knee | Ankle | Hip | Knee | Ankle |
| **×** | **√** | **√** | MAE | 0.019 | 0.092 | 0.018 | 0.16 | 0.14 | 0.14 | 5.12 | 5.59 | 3.78 |
|  |  |  | RMSE | 0.028 | 0.14 | 0.029 | 0.24 | 0.20 | 0.20 | 6.82 | 7.65 | 5.02 |
|  |  |  | *ρ* | 0.92 | 0.96 | 0.93 | 0.90 | 0.90 | 0.94 | 0.97 | 0.97 | 0.94 |
| **√** | **×** | **√** | MAE | 0.010 | 0.046 | 0.010 | 0.071 | 0.062 | 0.062 | 1.61 | 1.65 | 1.30 |
|  |  |  | RMSE | 0.017 | 0.077 | 0.019 | 0.11 | 0.11 | 0.10 | 2.31 | 2.42 | 1.95 |
|  |  |  | *ρ* | 0.97 | 0.99 | 0.97 | 0.97 | 0.97 | 0.99 | 0.997 | 0.998 | 0.99 |
| **√** | **√** | **×** | MAE | 0.010 | 0.044 | 0.010 | 0.071 | 0.062 | 0.062 | 1.45 | 1.43 | 1.27 |
|  |  |  | RMSE | 0.017 | 0.074 | 0.019 | 0.11 | 0.10 | 0.10 | 2.19 | 2.11 | 1.97 |
|  |  |  | *ρ* | 0.97 | 0.99 | 0.97 | 0.97 | 0.97 | 0.98 | 0.997 | 0.998 | 0.99 |
| **×** | **×** | **√** | MAE | 0.014 | 0.062 | 0.013 | 0.10 | 0.10 | 0.091 | 2.78 | 3.00 | 2.28 |
|  |  |  | RMSE | 0.021 | 0.10 | 0.023 | 0.16 | 0.15 | 0.14 | 4.04 | 4.30 | 3.29 |
|  |  |  | *ρ* | 0.95 | 0.98 | 0.96 | 0.95 | 0.94 | 0.97 | 0.99 | 0.99 | 0.98 |
| **√** | **√** | **√** | MAE | **0.0095** | **0.042** | **0.009** | **0.067** | **0.056** | **0.055** | **1.33** | **1.35** | **1.15** |
|  |  |  | RMSE | **0.016** | **0.074** | **0.019** | **0.11** | **0.10** | **0.091** | **1.98** | **2.10** | **1.77** |
|  |  |  | *ρ* | **0.97** | **0.99** | **0.97** | **0.97** | **0.97** | **0.99** | **0.997** | **0.998** | **0.99** |

**Supplementary References**

1. Delp SL, Anderson FC, Arnold AS, et al. OpenSim: open-source software to create and analyze dynamic simulations of movement. IEEE transactions on biomedical engineering 2007;54:1940–50.

2. Dembia CL, Bianco NA, Falisse A, Hicks JL, and Delp SL. OpenSim Moco: musculoskeletal optimal control. PLOS Computational Biology 2020;16:e1008493.

3. Wächter A, Biegler LT. On the implementation of an interior-point filter line-search algorithm for large-scale nonlinear programming[J]. Mathematical programming, 2006, 106(1): 25-57.
